# Adiponectin Regulated by Indole‐3‐Acetic Acid in Paneth Cells Controls Renewal and Differentiation of Gut Stem Cells

**DOI:** 10.1096/fj.202501229RR

**Published:** 2025-10-06

**Authors:** Hang Liu, Xiaomin Su, Juanjuan Wang, Mengli Jin, Yuan Zhang, Rongcun Yang

**Affiliations:** ^1^ Department of Immunology Nankai University School of Medicine, Nankai University Tianjin China; ^2^ State Key Laboratory of Medicinal Chemical Biology Nankai University Tianjin China; ^3^ Translational Medicine Institute Tianjin Union Medical Center of Nankai University Tianjin China; ^4^ Key Laboratory of Bioactive Materials Ministry of Education Nankai University Tianjin China

**Keywords:** adiponectin, gut epithelial cells, indole‐3‐acetic acid, intestinal stem cells, Paneth cells

## Abstract

The intestinal epithelium is tightly regulated by intestinal stem cells (ISCs), but the precise mechanisms governing their differentiation remain incompletely understood. We here demonstrate that adiponectin secreted by Paneth cells (PCs) suppresses ISC renewal and differentiation via adiponectin receptor 1 (adipoR1). Genetic ablation of adiponectin in gut epithelial cells (adip^fl/fl‐Villi‐Cre^ mice) enhanced crypt cell renewal and differentiation. Single‐cell RNA sequencing (scRNA‐seq) revealed a significant increase in the enrichment of ISCs and transit‐amplifying (TA) cells in adip^fl/fl‐Villi‐Cre^ mice compared to control adip^fl/fl^ mice. Furthermore, adip^fl/fl/‐Villi‐Cre^ mice exhibited accelerated regeneration of intestinal epithelial cells following irradiation or dextran sulfate sodium (DSS)‐induced injury. Intestinal organoids derived from adip^fl/fl‐Villi‐Cre^ mice also displayed markedly faster growth than those from adip^fl/fl^ mice. Consistent with these findings, adipoR1 knockout (KO) mice exhibited elongated crypt structures, further supporting adiponectin's inhibitory role in ISC proliferation. Notably, gut microbiota‐derived indole‐3‐acetic acid (IAA) downregulated adiponectin expression, thereby promoting ISC renewal and proliferation. This was corroborated by in vitro organoid cultures, where IAA treatment accelerated development. Thus, our findings reveal that adiponectin modulated by microbial IAA serves as a critical regulator of ISC dynamics, ensuring epithelial homeostasis.

## Graphical abstract

The adiponectin produced by Paneth cells suppresses intestinal stem cell renewal and differentiation by signaling through adiponectin receptor 1. This inhibitory effect is counterbalanced by gut microbiota‐derived indole‐3‐acetic acid, which activates the pregnane X receptor to downregulate adiponectin expression, consequently enhancing intestinal stem cell renewal and proliferation.

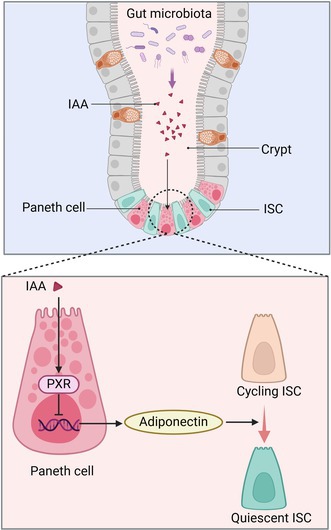

## 1 Introduction

The gut epithelium consists of a single layer of cells that form crypt‐villus units, the fundamental structural and functional components of the intestinal lining [1]. This epithelial layer comprises multiple specialized cell types, including enterocytes (ECs), goblet cells (GCs), tuft cells, enteroendocrine cells (EECs), stem cells, and Paneth cells (PCs). Anatomically, crypts represent epithelial invaginations at the base of villi [1], while villi are finger‐like projections extending into the intestinal lumen. Intestinal stem cells (ISCs), located at the crypt base and interspersed among PCs, play a pivotal role in maintaining epithelial homeostasis. The intestinal epithelium undergoes complete renewal every 3–5 days, a process rigorously regulated by ISCs [2]. These stem cells generate highly proliferative transit‐amplifying (TA) cells, which subsequently differentiate into all epithelial lineages along the crypt‐villus axis [3]. Notably, gut stem cells possess the remarkable capacity to self‐organize into crypt‐villus structures even in vitro [4].

Gut stem cell renewal and differentiation are regulated by intricate epithelial and stromal signaling networks [5]. The ISC niche comprises multiple supporting cell types, including pericryptal myofibroblasts, fibroblasts, pericytes, endothelial cells, PCs, immune cells, neural cells, and smooth muscle cells. These cells secrete diverse extracellular matrix components and growth factors that critically modulate ISC self‐renewal and differentiation [6]. PCs, located at the crypt base, play a pivotal role in maintaining the ISC niche. They provide essential niche signals, such as Wnt3, EGF, TGF‐α, and Dll4, to sustain Lgr5^+^ stem cell proliferation [5]. The crypt region harbors both quiescent and actively cycling ISCs, highlighting the dynamic regulation of stem cell behavior. Notably, certain immune‐derived factors, including Th17 and Th2 cytokines, can also suppress stem cell renewal and differentiation [7]. Despite these advances, the complete regulatory mechanisms governing ISC fate decisions remain incompletely understood.

Adiponectin is a multifunctional protein produced not only by adipose tissue and macrophages but also by epithelial cells [8, 9]. It exhibits broad protective effects against obesity, insulin resistance, inflammation, and cardiovascular diseases [10]. Through binding to its receptors (adipR1 and adipR2) [11], adiponectin activates multiple signaling pathways, including AMP‐activated protein kinase (AMPK). Intriguingly, AMPK activation has been shown to inhibit several proliferative signaling pathways involved in the renewal and proliferation of ISCs and other epithelial lineages [12, 13]. Consistent with this mechanism, accumulating evidence indicates that adiponectin exerts suppressive effects on colorectal carcinogenesis [14, 15], and adiponectin receptor agonists have demonstrated potent anti‐tumor activity in various models [16, 17, 18]. Notably, an inverse correlation has been observed between adiponectin levels and the expression of stemness factors in tumor cells [19]. Of particular note, our previous study identified adiponectin expression in cells located at the base of intestinal crypts [9, 20], suggesting a potential autocrine/paracrine role for adiponectin in regulating intestinal stem cell dynamics and epithelial differentiation. Herein, we demonstrate that adiponectin expression in PCs plays a crucial role in regulating intestinal stem cell renewal and differentiation, thereby maintaining intestinal epithelial homeostasis. Previous studies have established that the cellular architecture of the intestinal epithelium is modulated by gut microbiota in both community‐ and species‐specific manners [21, 22]. Our current findings reveal that indole‐3‐acetic acid (IAA), a microbial‐derived metabolite, modulates adiponectin expression in PCs, which in turn orchestrates the balance between stem cell renewal and differentiation in the intestinal epithelium.

## 2 Materials and Methods

The reagents and oligos used in this study are listed in Table S1.

### 2.1 Mice

Four‐ to six‐week‐old male and female C57BL/6 mice were obtained from the Nanjing Animal Center. Gut epithelial cell‐specific adiponectin conditional knockout mice (adip^fl/fl‐Villi‐Cre^) and their control counterparts (adip^fl/fl^) were generated by GemPharmatech Co. Ltd. (Nanjing, China). Adiponectin receptor 1 (adipR1) and receptor 2 (adipR2) knockout mice were developed by the Animal Center of Nankai University. Additional genetically modified strains included Lgr5EGFP‐IRES‐creERT2 mice (provided by Beijing Agricultural University, Beijing, China), *pVilliCreT* mice (generated by GemPharmatech), aryl hydrocarbon receptor (AHR) knockout mice (obtained from the Third Military Medical University, Chongqing, China), pregnane X receptor (PXR) and constitutive androstane receptor (CAR) knockout mice (provided by the Chinese Academy of Inspection and Quarantine, Tianjin, China). All mice were maintained under specific pathogen‐free (SPF) conditions in the Animal Center of Nankai University. Age‐ and gender‐matched littermates were used as controls in all experiments. Animal experiments were approved by the Institutional Animal Care and Use Committee of Nankai University (Approval No. 2019‐SYDWLL‐000651) and conducted in compliance with the guidelines of the Model Animal Research Center. Strict environmental and husbandry controls were implemented throughout the study.

### 2.2 Mouse Models

For the ionizing radiation (IR) mouse model, we followed the previously established experimental procedure [23]. Briefly, experimental mice were subjected to γ‐irradiation at a total dose of 10 Gy (dose rate: 1.2 Gy/min) using a Shepherd Mark I Cesium Irradiator (J.L. Shepherd and Associates). Control mice were placed in the irradiator under identical conditions but without radiation exposure. Mice were randomly allocated to either the control group or the IR group. Intestinal epithelial injury was assessed according to the following histopathological grading scale [24], Grade 0: Normal mucosal villi architecture; Grade 1: Subepithelial cystic spaces and capillary hyperemia restricted to villus apices; Grade 2: Expanded subepithelial cystic spaces with edema extending into the lamina propria and dilation of central lacteals; Grade 3: Epithelial cell degeneration/necrosis, severe lamina propria edema, and focal villus tip denudation; Grade 4: Extensive epithelial cell degeneration/necrosis/exfoliation, capillary hyperemia/dilation, lamina propria exposure, and partial villus denudation; Grade 5: Hemorrhage, ulceration, lamina propria disintegration, and complete villus loss.

DSS‐induced colitis was established as previously described [25]. Briefly, experimental mice were administered 2.5% DSS (40 000 kDa; MP Biomedicals) in their drinking water for 7 days, followed by a return to regular drinking water. Histological scoring was performed according to established criteria [26, 27]. Histology was scored as follows: epithelium (E), 0 = normal morphology; 1 = loss of goblet cells; 2 = loss of goblet cells in large areas; 3 = loss of crypts; 4 = loss of crypts in large areas; and infiltration (I), 0 = no infiltrate; 1 = infiltrate around the crypt basis; 2 = infiltrate reaching the lamina (L) muscularis mucosae; 3 = extensive infiltration reaching the L muscularis mucosae and thickening of the mucosa with abundant oedema; 4 = infiltration of the L submucosa. Total histological score was given as E + I. Mice were treated with IAA at a dose of 100 mg/kg via oral gavage daily for 7 days before subsequent experiments. The bacterial transplantation protocol was adapted from a previously reported method [28]. Before transplantation, mice received a pan‐antibiotic cocktail in their drinking water, consisting of ampicillin (1 g/L, Sigma), vancomycin (0.5 g/L, Sigma), neomycin sulfate (1 g/L, Sigma), and metronidazole (1 g/L, Sigma). The antibiotic solution was refreshed every 3 days. To verify bacterial depletion, fecal samples were collected and cultured under both anaerobic and aerobic conditions. For transplantation, *Lactobacillus* strains were suspended in PBS containing 30% glycerol at a concentration of 1 × 10^9^ CFU/mL, and 200 μL of the bacterial suspension was administered orally.

### 2.3 Single‐Cell RNA‐Seq Processing

For preparation of single‐cell suspensions, obtained tissues were transferred to a Petri dish pre‐spiked with 1 × PBS (without RNase and Ca, Mg ions) and washed with 1× PBS to remove blood stains, grease, and other adherents from the tissue surface. The tissue was then cut into 0.5 mm 2 pieces and washed again with 1 × PBS. The washed pieces were added with dissociation solvent (0.35% collagenase IV5, 2 mg/mL papain, 120 Units/ml DNase I) and reacted for 20 min at 37°C in a water bath shaker (100 rpm). The cells were repeatedly pipetted up and down 5–10 times with a pipette gun. The cell suspension was filtered through a 70 μm cell sieve and subsequently centrifuged at 300 g for 5 min at 4°C. After centrifugation, the cell sediment was collected, and the cells were resuspended by adding 100 μL of 1× PBS (0.04% BSA) solution. To remove erythrocytes, 1 mL of 1× erythrocyte lysis solution (MACS 130–094‐183, 10×) was added and reacted at room temperature or on wet ice for 2–10 min. After lysis, the cells were centrifuged at 300 g for 5 min at 4°C, and the cell precipitate was collected after centrifugation. Add 100 μL of Dead Cell Removal MicroBeads (MACS,130‐090‐101), mix well, and incubate for 15 min at room temperature. At the end of incubation, dead cells were removed after MS separation columns (130‐042‐201). Cell precipitates were collected by centrifugation at 300 *g* for 5 min at 4°C. Subsequently, 1 × PBS (0.04% BSA) was used to resuspend the cell precipitate and centrifuged at 4°C 300 *g* for 5 min (repeated twice). The cell activity was detected by trypan blue staining method, and the cell activity was required to be > 85%. The number of cells was counted using a hemocytometer or Countess II Automated Cell Counter, and the cell concentration was 700–1200 cells/μL.

For chromium 10× Genomics library and sequencing, single‐cell suspension was added to the 10× Chromium chip according to the instructions for the 10× Genomics Chromium Single‐Cell 3′ kit (V3), with the expectation of capturing 8000 cells. cDNA amplification and library construction were performed according to standard protocols. Libraries were sequenced by LC‐Bio Technology (Hangzhou, China) on an Illumina NovaSeq 6000 sequencing system (double‐end sequencing, 150 bp) at a minimum depth of 20 000 reads per cell.

For bioinformatics analysis, results from Illumina sequencing were converted to FASTQ format using bcl2fastq software (version 5.0.1). The scRNA‐seq sequencing data were compared to the reference genome using CellRanger software, and cellular and individual cellular 3′ end transcripts were identified and counted in the sequenced samples (https://support.10xgenomics.com/single‐cell‐gene expression/software/pipelines/latest/what‐is cell‐ranger, version 7.0.0). The output CellRanger expression profile matrix was loaded into Seurat (version 4.1.0) for filtering low‐quality cells from scRNA‐seq data, and the filtered data were downscaled and clustered. Filtering low cell quality thresholds: Number of genes expressed per cell > 500, mitochondrial genes expressed in < 25% of cells. Cells were projected into 2D space using t‐SNE or UMAP. These steps include calculating gene expression values using the LogNormalize method of Seurat's “Normalize Data” function; performing principal component analysis (PCA) using the normalized expression values, using the top 20 PCs for clustering and FindCluster analysis; analyzing the marker genes of each cluster based on FindAllMarkers. The marker genes were selected based on the following criteria: expressed in more than 10% of cells in each cluster, P value ≤ 0.01, gene expression ploidy logFC ≥ 0.26; hypergeometric testing was used to perform GO and KEGG enrichment analysis on the differential genes of each cluster obtained from FindAllMarkers analysis relative to other clusters. SingleR database, scCATCH database, and our self‐developed LC‐Marker were used to identify each cluster cell type, and some cells were re‐clustered based on the identification results of the SingleR database to find marker genes, cell communication analysis, and pseudotime analysis to provide ideas for further analysis. For plotting, violin plots and feature plots were created using the VlnPlot function and FeaturePlot function in Seurat V4. The DimPlot function in Seurat V4 was used to graph the output of the dimensional reduction on a 2D scatter plot. The DoHeatmap function in Seurat V4 was used to draw the heatmap of single‐cell feature expression.

### 2.4 Isolation of Crypts, in Vitro Cultured Organoids, and Sorting of Stem Cell and Paneth Cells

Crypt isolation was performed as previously described [29]. Briefly, intestinal or colonic tissues were dissected, flushed with buffer, and cut into small segments. Villi were mechanically scraped before crypt isolation. Tissue fragments were incubated in crypt isolation buffer (1.5 mM KCl, 96 mM NaCl, 27 mM sodium citrate, 8 mM KH₂PO₄, 5.6 mM Na₂HPO₄, 15 mM EDTA) for 15 min at 4°C to release crypts. Intestinal organoid cultures were established following established protocols [4, 30]. Isolated crypts were suspended in Matrigel and plated in 24‐well plates. Cultures were maintained in DMEM/F12‐ENR medium, with medium changes performed every 48 h. Mature small intestinal organoids (7‐day culture) or colonic organoids (9‐day culture) were treated with 100 μM IAA for 24 h. For stem cell and PC isolation, organoids were enzymatically dissociated using TrypLE Express Enzyme (Thermo Fisher Scientific) for 15 min followed by 0.2 mg/mL DNase I (Roche) treatment for 10 min at 37°C with agitation. The resulting cell suspension was filtered through a 40 μm strainer (Biosharp), washed with PBS, and stained with anti‐mouse LGR5 antibodies for subsequent fluorescence‐activated cell sorting (FACS).

### 2.5 Hematoxylin and Eosin Staining

Hematoxylin and eosin (H & E) staining was performed using established protocols [31]. Briefly, the entire intestine or colon was excised, fixed in 4% (w/v) paraformaldehyde‐buffered saline, and embedded in paraffin. Tissue sections (5 μm thick) were then prepared and stained with H&E. For crypt length analysis, intact crypts from mice were measured using Photoshop CS5, and their relative lengths were calculated using wild‐type (WT) mice as a reference. For quantification of Ki67‐positive cells, complete crypt‐villus units were scaled using Adobe Illustrator, and the number of Ki67‐positive cells was counted.

### 2.6 Immune Staining

For in vitro organoid staining, we followed established protocols [30]. Briefly, organoids were treated with dispase to partially dissolve Matrigel, gently collected by centrifugation, and blocked with 1× blocking buffer. Primary and secondary antibody incubations were performed sequentially, followed by nuclear counterstaining with DAPI. For intestinal immune staining [30], mouse gut tissues were fixed, paraffin‐embedded, and sectioned into 5‐μm slices. Sections were immunostained using primary antibodies followed by appropriate fluorescent secondary antibodies.

### 2.7 RNA‐Seq Analysis

The expression profiles of genes in in vitro cultured gut organoids were analyzed using RNA sequencing (RNA‐seq), and performed by BGI in Wuhan, China. Total RNA was extracted from the cells following the manufacturer's protocol for TRIzol reagent (Invitrogen, Shanghai, China). Sequencing libraries were prepared and transcriptome analysis was conducted on the MGISEQ‐2000 platform, generating 100 bp paired end reads. Read alignment and quantification were performed using the MGISEQ‐2000 system, with gene expression levels calculated as fragments per kilobase of transcript per million mapped fragments (FPKM). Differential gene expression was assessed based on fold change (FC) values. Data analysis was carried out using the bioinformatics tools available on the BGI analysis platform (http://biosys.bgi.com).

### 2.8 Western Blot, QRT‐PCR, and ELISA

Previously reported Western blot, QRT‐PCR, and ELISA [28, 31] were used in this study.

### 2.9 Statistics

Statistical analyses were performed using the two‐tailed Student *t* test and Mann–Whitney U test. These were performed using GraphPad Prism 7 software. A 95% confidence interval was considered significant and was defined as *p < 0.05; **p < 0.01; ***p < 0.001.

## 3 Results

### 3.1 Expressions of Adiponectin in Gut Paneth Cells

**FIGURE 1:**
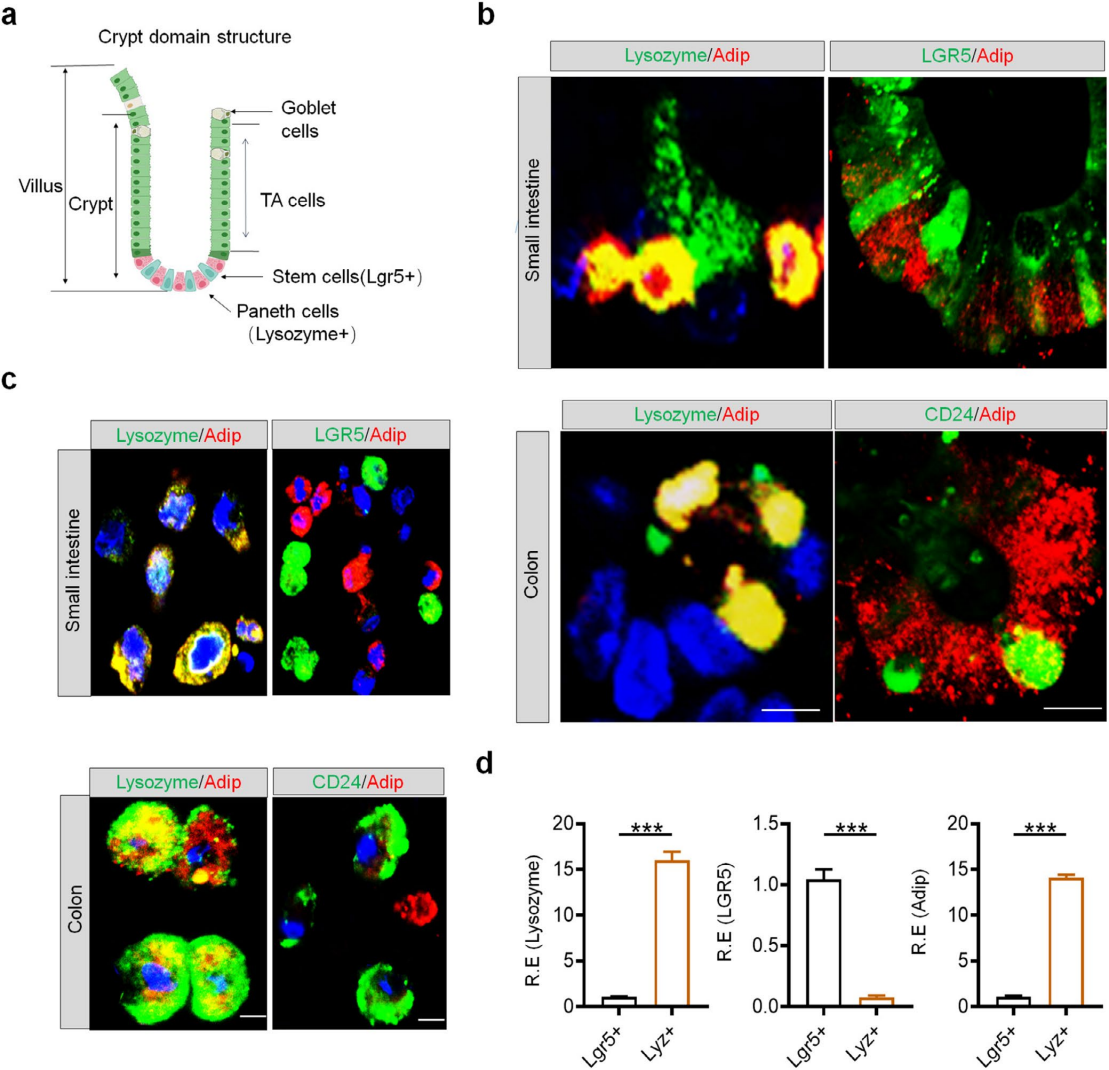
Adiponectin expression in Paneth cells of intestinal and colonic epithelial cells. (a) A schematic illustration showing the structure of the crypt domain in the intestinal villi. TA，transit amplifying; (b) Immunostaining of adiponectin (Adip, red) and lysozyme (Paneth cells, green) or Lgr5 (Intestinal stem cell, green) and CD24 (Colon stem cell, green) in the crypts of small intestinal and colonic tissues. Scale bar = 10 μm. A representative from three mice. (c) Immunostaining of adiponectin (Adip, red) and lysozyme (Paneth cells, green) or Lgr5 (Intestinal stem cell, green) and CD24 (Colon stem cell, green) in the isolated cells from in vitro cultured intestinal or colonic organoids. Scale bar = 25 μm in intestine; Scale bar = 10 μm in colon. (d) QRT‐PCR of lysozyme, lgr5, and adiponectin (Adip) in sorted intestinal stem cells (lgr5^+^ cells) and Paneth cells (Lyz^+^) from in vitro cultured intestinal by flow cytometry. Student's *t*‐test, mean ± SD; ***p < 0.001. R. E., relative expression.

Adiponectin expression is predominantly localized to the crypt epithelium [9, 32, 33], with specific enrichment in PCs [33]. Building upon prior reports [33], our immunohistochemical analysis revealed robust adiponectin expression in Lyz^+^ PCs, while being undetectable in either Lgr5^+^ ISCs or CD24^+^ colonic stem cells (Figure 1a,b). We also performed single‐cell analysis of dissociated intestinal and colonic organoids to further characterize adiponectin‐expressing populations. This comprehensive approach unequivocally demonstrated adiponectin expression exclusively in Lyz^+^ PCs, with no detectable expression in other intestinal epithelial cell lineages derived from organoid cultures (Figure 1c). QRT‐PCR analysis of sorted cell populations provided independent validation of adiponectin expression specifically in PCs (Figure 1d). Taken together, these findings establish that adiponectin is constitutively expressed by canonical PCs in the small intestine and their Paneth‐like counterparts in the colon.

### 3.2 Adiponectin Gut Conditional Knockout Promotes Recovery of Damaged Gut Epithelial Cells

**FIGURE 2:**
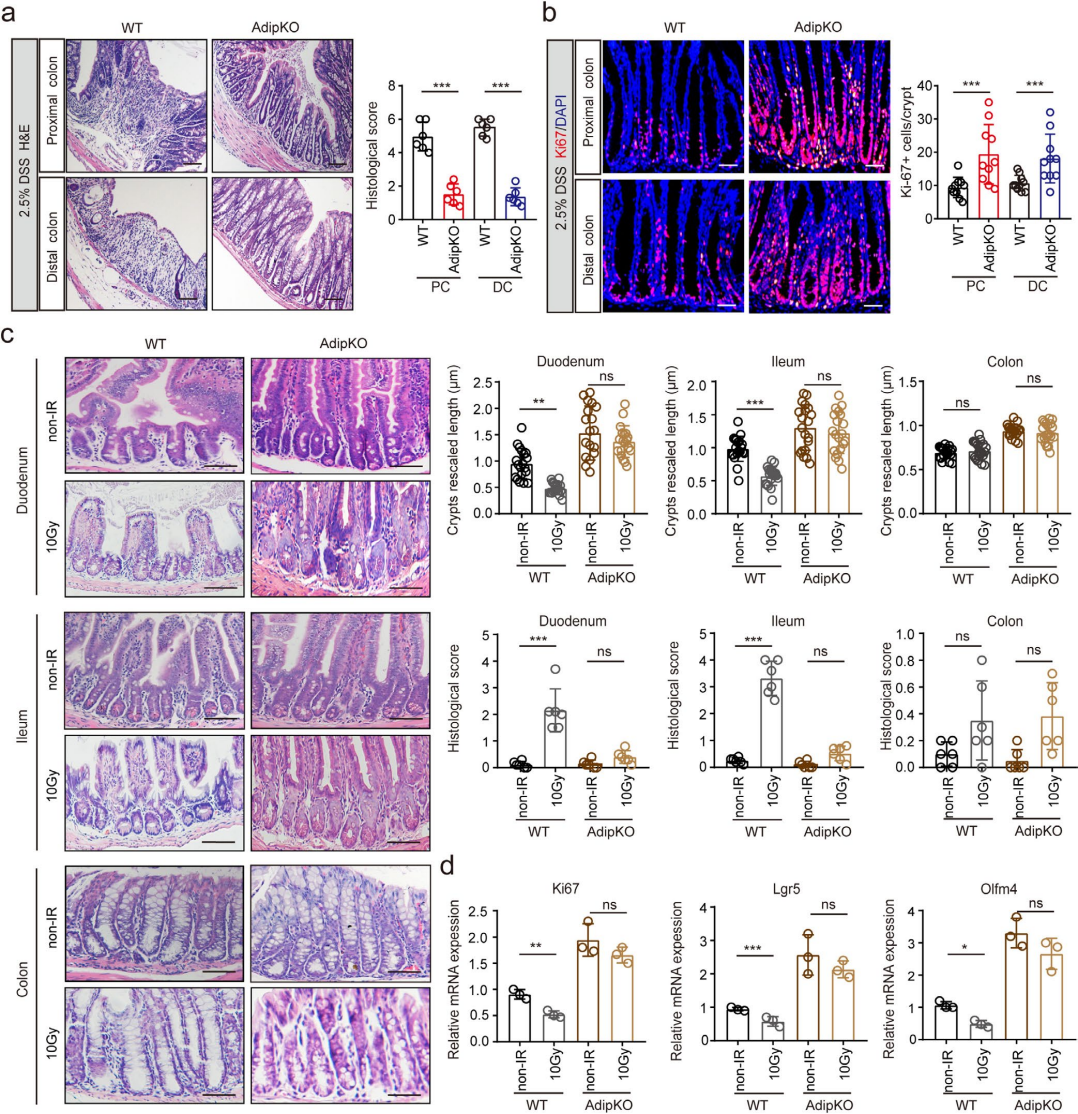
Adiponectin deficiency promotes repair of gut epithelial damage. (a) H&E staining of the proximal and distal colonic tissue of adip^fl/fl‐Villi‐Cre^ (AdipKO) and adip^fl/fl^ (WT) mice at 7‐day post start of DSS (2.5%) treatment. Scale bar = 45 μm. (b) Immunostaining of Ki67 in the proximal and distal colonic tissue of adip^fl/fl‐Villi‐Cre^ (AdipKO) and adip^fl/fl^ (WT) mice at 12‐day post start of DSS (2.5%) treatment. *n* = 102 crypt‐villus units from 5 mice per group. Scale bar = 45 μm. (c) H&E staining of the duodenum and ileum, and colon tissue in adip^fl/fl‐Villi‐Cre^ (AdipKO) and adip^fl/fl^ (WT) mice after irradiation (10Gy). *n* = 105 crypts from 6 mice per group. Scale bar = 45 μm. (d) qRT‐PCR of ki67, lgr5, and olfm4 in gut tissues of adip^fl/fl‐Villi‐Cre^ (AdipKO) and adip^fl/fl^ (WT) mice after irradiation (10 Gy). At day 3 after radiation, intestinal tissue was collected. Mann–Whitney U test in a, b, and c; Student's *t*‐test in d, mean ± SD; *p < 0.05, **p < 0.05, ***p < 0.05; Ns, no significance.

Adiponectin has been demonstrated to suppress colorectal carcinogenesis [14, 15]. Based on this evidence, we postulated that adiponectin secreted by intestinal PCs might exert paracrine effects on neighboring ISCs within the crypt niche, thereby modulating the regenerative capacity and proliferative activity of intestinal epithelial cells. To investigate this hypothesis, we used two well‐established injury models, ionizing radiation [34] and DSS‐induced colitis [35] to systematically evaluate the functional contribution of PC‐derived adiponectin in epithelial repair processes. We generated adiponectin conditional knockout mice (adip^fl/fl‐Villi‐Cre^) and their corresponding control littermates (adip^fl/fl^) (Figure S1). Following a 7‐day administration of 2.5% (wt/vol) DSS in drinking water and subsequent recovery period with normal water, adip^fl/fl‐Villi‐Cre^ mice demonstrated significantly enhanced intestinal epithelial regeneration compared to controls (Figure 2a). This accelerated regenerative response was associated with a pronounced increase in epithelial cell proliferation, as evidenced by quantitative analysis of Ki67‐positive cells (a well‐validated proliferation marker [36]), along with improved architectural restoration of the epithelial lining in adip^fl/fl‐Villi‐Cre^ mice (Figure 2b).

We next evaluated the regenerative capacity of the intestinal epithelium by subjecting adip^fl/fl‐Villi‐Cre^ mice and adip^fl/fl^ control mice to γ‐irradiation (10 Gy). Histopathological examination (H&E staining) combined with immunohistochemical analysis demonstrated markedly enhanced proliferation and tissue regeneration in the small intestine and colon of adip^fl/fl‐Villi‐Cre^ mice as compared to controls (Figure 2c). Quantitative measurements revealed significantly elongated crypt lengths in irradiated adip^fl/fl‐Villi‐Cre^ mice (Figure 2c), indicative of heightened regenerative activity. Consistent with these morphological observations, molecular analysis showed substantially elevated expression levels of proliferation‐associated markers (Ki67, Lgr5, Olfm4 [37]) in intestinal epithelial cells of adip^fl/fl‐Villi‐Cre^ mice at day 3 post‐irradiation (Figure 2d). These collective findings provide compelling evidence that targeted depletion of adiponectin in gut PCs specifically augments the proliferative and regenerative potential of intestinal epithelial cells following radiation‐induced injury.

### 3.3 Adiponectin Deficiency in Paneth Cells Promotes the Development of Gut Stem Cells

**FIGURE 3:**
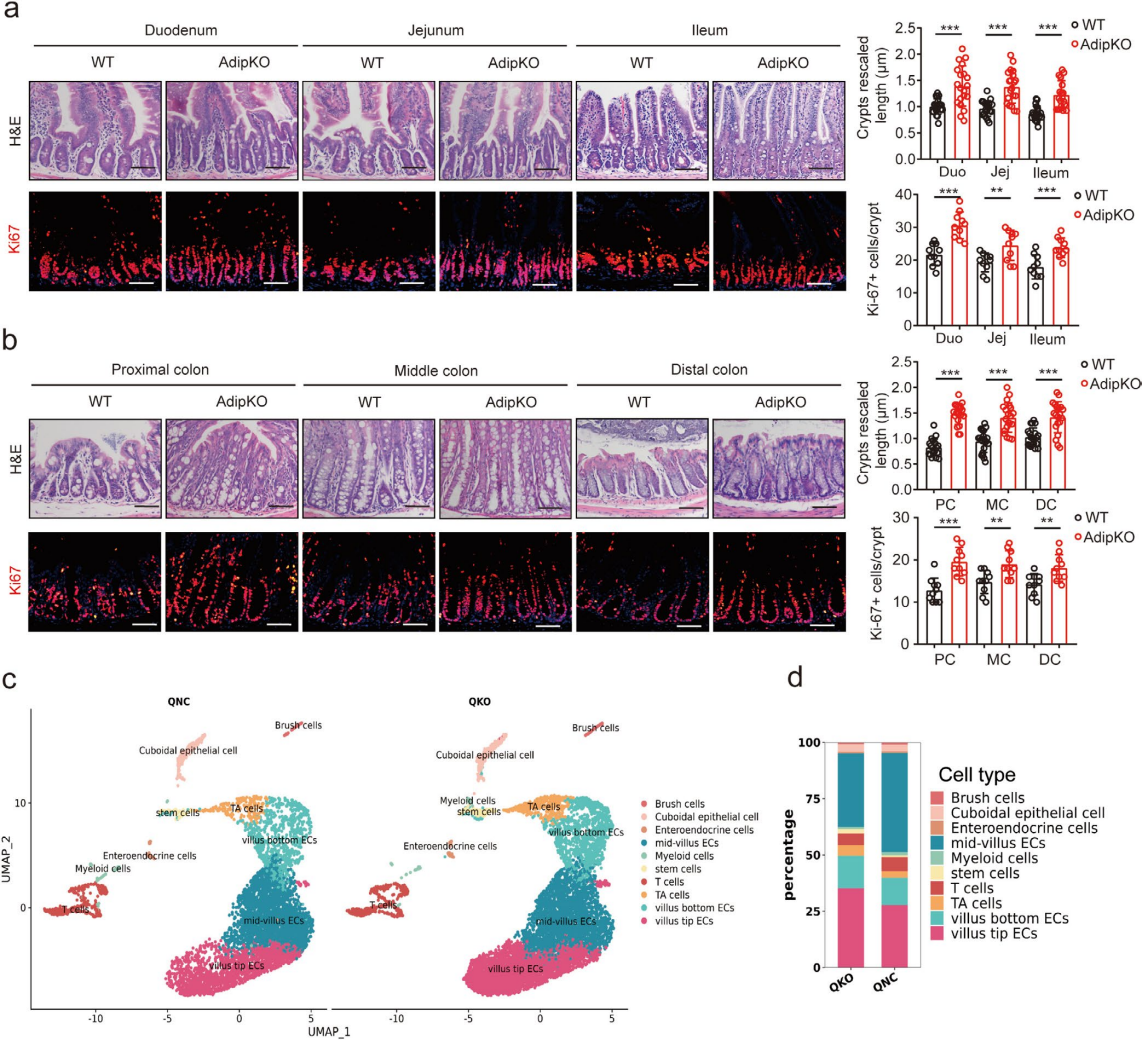
Adiponectin regulates the proliferation and differentiation of gut stem cells. (a) H/E staining and immunostaining of Ki67(red) in the small intestinal (duodenum, jejunum, and ileum) tissues of adip^fl/fl‐Villi‐Cre^ (AdipKO) and adip^fl/fl^ (WT) mice. *n* = 91 crypts from 5 mice per group for crypt rescaled length; *n* = 62 crypt‐villus units from 5 mice per group for Ki67 cells; Scale bar = 45 μm; (b) H/E staining and immunostaining of Ki67(red) in colonic (proximal colon, middle colon, and distal colon) tissues of adip^fl/fl‐Villi‐Cre^ (AdipKO) and adip^fl/fl^ (WT) mice. DAPI, nuclei (blue). *n* = 91 crypts from 5 mice per group for crypt rescaled length; *n* = 62 crypt‐villus units from 5 mice per group for Ki67 cells; Scale bar = 45 μm; (c) UMAPs of scRNA‐seq in the intestinal tissue of adip^fl/fl‐Villi‐Cre^ (AdipKO) and their littermate adip^fl/fl^ (WT) mice. Pooled sample from 8‐week‐old male mice, *n* = 6. (d) Percentages of different cell clusters in the intestinal tissue of adip^fl/fl‐Villi‐Cre^ (AdipKO) and adip^fl/fl^ (WT) mice. Mann–Whitney U test in a and b; **p < 0.05, ***p < 0.05.

To investigate the role of adiponectin derived from gut PCs, we analyzed ISCs and their adjacent TA cells in both adip^fl/fl‐Villi‐Cre^ mice and control adip^fl/fl^ mice. Genetic ablation of adiponectin in gut epithelial cells significantly promoted the expansion of ISCs and TA cells within intestinal crypts. Compared to control mice, adip^fl/fl‐Villi‐Cre^ mice exhibited markedly enlarged crypts that were predominantly populated by these proliferative cell populations (Figure 3a,b). This morphological alteration clearly demonstrated enhanced enrichment of both ISCs and TA cells in the crypts of adip^fl/fl‐Villi‐Cre^ mice. Furthermore, we observed a substantial increase in Ki67‐positive proliferating cells throughout the crypt‐villus axis in adip^fl/fl‐Villi‐Cre^ mice (Figure 3a,b). Collectively, these findings demonstrate that PC‐derived adiponectin plays a critical role in regulating the homeostasis and proliferation of intestinal crypt cells, particularly ISCs and TA cells.

To further elucidate the effects of PC‐derived adiponectin on ISCs and TA cells, we conducted single‐cell RNA sequencing (scRNA‐seq) on crypt‐enriched small intestinal epithelium from both control adip^fl/fl^ mice and adip^fl/fl‐Villi‐Cre^ mice. Crypt enrichment was performed to enhance the capture of ISCs and TA cells while maintaining representation of all epithelial cell populations along the crypt‐villus axis [38]. Initial sequencing yielded 21 629 cells, with 9791 cells from adip^fl/fl^ mice and 11 838 cells from adip^fl/fl‐Villi‐Cre^ mice. Following quality control filtering, we retained 6757 high‐quality cells from adip^fl/fl^ mice and 8550 from adip^fl/fl‐Villi‐Cre^ mice for subsequent analysis. Unsupervised clustering identified 14 distinct epithelial cell populations, which were annotated as ISCs, TA cells, and other intestinal epithelial subtypes based on established marker genes [39, 40, 41] (Figure 3c; Figure S2). To explore potential adiponectin‐mediated effects on epithelial differentiation, we performed trajectory inference analysis. Although there revealed similar trajectories from ISCs through TA cells to mature epithelial lineages in both genotypes (Figure 3c), quantitative analysis of cell population distributions demonstrated significant increases in the proportions of both ISCs and TA cells in adip^fl/fl‐Villi‐Cre^ mice as compared to controls (Figure 3d). These findings indicate that adiponectin deficiency in PCs enhances the proliferation and differentiation of ISCs and TA cells. Thus, our results demonstrate that adiponectin produced by PCs serves as a negative regulator of intestinal stem cell renewal and TA cell differentiation.

### 3.4 AdipR1 KO Promotes the Proliferation of Gut Stem Cells

**FIGURE 4:**
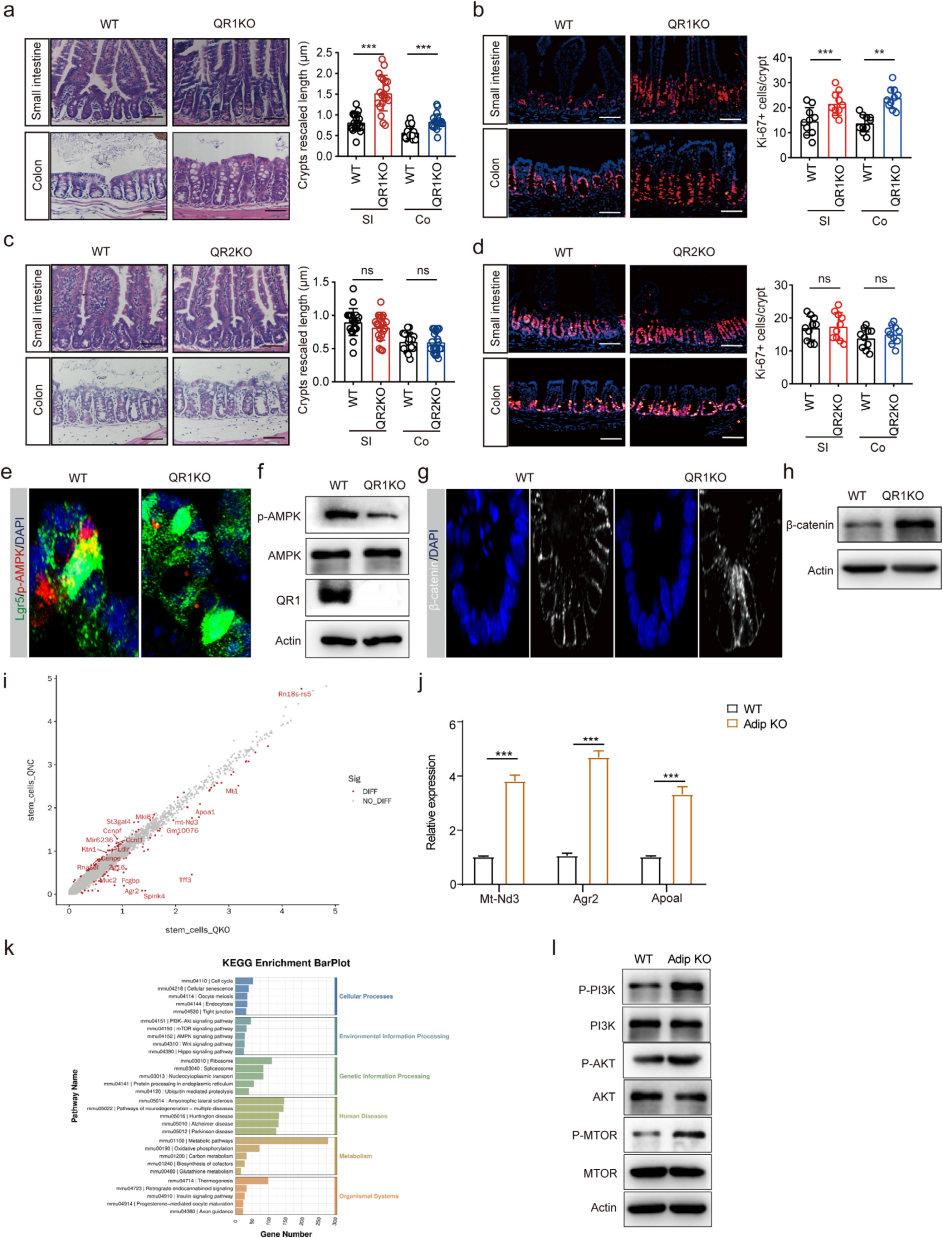
Adiponectin receptor 1 knockout in mice promotes proliferation of gut stem cells. (a) H&E staining of the small intestine and colon tissues of adipR1 KO (QR1KO) and WT mice. *n* = 105 crypts from 5 mice per group. Scale bar = 45 μm; (b) Immunostaining of Ki67 in the small intestine and colon tissues of adipR1 KO (QR1KO) and WT mice. DAPI for cell nuclei (blue). *n* = 66 crypt‐villus units from 5 mice per group. Scale bar = 45 μm; (c) H&E staining of the small intestine and colon tissues of adipR 2 KO (QR2KO) and WT mice. *n* = 84 crypts from 5 mice per group. Scale bar = 45 μm; (d) Immunostaining of Ki67 in the small intestine and colon tissues of adipR2KO (QR2KO) and WT mice. DAPI, nuclei (blue). *n* = 60 crypt‐villus units from 5 mice per group; (e) Immunostaining of Lgr5 (green)/p‐AMPK(red) in the ileum crypts of adipR1 KO (QR1KO) and WT mice; (f) Immunoblot of p‐AMPK and AMAK in the ileum crypts of adipR1 KO (QR1KO) and WT mice; Actin as the loading control; (g) Immunostaining of β‐catenin (gray) in the ileum crypts of adipR1 KO (QR1KO) and WT mice; (h) Immunoblot of β‐catenin in the ileum crypts of adipR1 KO (QR1KO) and WT mice; Actin as the loading control; (i) Gene expression of intestinal stem cell cluster by scRNA sequence in adip^fl/fl‐Villi‐Cre^ (QKO) and adip^fl/fl^ (QNC) mice; (j) QRT‐PCR of mt‐Nd3, agr2, and apoal in the isolated ileum crypts of adip^fl/fl‐Villi‐Cre^ (QKO) and adip^fl/fl^ (QNC) mice. (k) KEGG analyses of intestinal stem cell cluster by scRNA sequence in adip^fl/fl‐Villi‐Cre^ (QKO) and adip^fl/fl^ (QNC) mice. (l) Immunoblotting of pPI3K, PI3K, P‐AKT, AKT, pmTOR and mTOR in the isolated ileum crypts of adip^fl/fl‐Villi‐Cre^ (AdipKO) and their littermate adip^fl/fl^ (WT) mice. Student's *t*‐test in j, mean ± SD; Mann–Whitney U test in a, b, c, and d; **p < 0.05, ***p < 0.05; Ns, no significance.

We next sought to elucidate the mechanism by which adiponectin regulates the proliferation of Lgr5^+^ ISCs. Adiponectin primarily signals through its cognate receptors adipR1 and adipR2, both of which are expressed in normal intestinal epithelium and colorectal cancer tissues [42, 43] (Figure S3). To delineate the specific functions of these receptors, we generated adipR1‐ and adipR2‐deficient mouse models. Intriguingly, histological analysis revealed a significant increase in crypt cell proliferation specifically in adipR1 knockout mice, while adipR2 deficiency showed no such effect (Figure 4a–d; Figure S4), suggesting that adiponectin mediates its proliferative effects predominantly through adipR1 signaling. Mechanistically, adiponectin‐receptor engagement typically triggers tissue‐specific downstream signaling cascades, including AMP‐activated protein kinase (AMPK) activation [44]. Consistent with this paradigm, we observed diminished AMPK phosphorylation in the ileum crypts of adipR1 knockout mice (Figure 4e,f). Notably, levels of β‐catenin, the central effector of the Wnt/β‐catenin pathway that governs intestinal stem cell maintenance and proliferation [45], were markedly elevated in the ileum crypts of adipR1‐deficient mice (Figure 4g,h).

Adiponectin‐activated AMPK is known to inhibit multiple signaling pathways [12, 13]. To investigate its role in gut crypt development, we performed differential gene expression analysis on scRNA‐seq data comparing control *adip* ^*fl/fl*^ mice and *adip* ^*fl/fl‐Villi‐Cre*^ mice. Notably, stem cells from *adip* ^*fl/fl‐Villi‐Cre*^ mice exhibited elevated expression of stem cell marker genes (e.g., mt‐Nd3, *agr2*, and *apoa1*) (Figure 4i,j). KEGG pathway enrichment analysis further revealed enhanced proliferative and metabolic activity in these stem cells, characterized by upregulation of PI3K‐Akt–mTOR, Wnt, and Hippo signaling pathways, along with key metabolic pathways (Figure 4k,l). These findings suggest a diminished AMPK‐mediated suppression of these signaling cascades in the absence of adiponectin. Similarly, TA cells from *adip* ^*fl/fl‐Villi‐Cre*^ mice displayed not only increased expression of TA marker genes (mt‐Nd3, *sec61g*) but also activation of PI3K‐Akt–mTOR and cGMP‐PKG signaling pathways (Figure S5), both of which play pivotal roles in maintaining cellular homeostasis [46, 47]. Thus, our results demonstrate that adiponectin regulates gut crypt cell development via adipoR1, primarily through AMPK‐dependent suppression of key signaling pathways such as PI3K‐Akt–mTOR.

### 3.5 Adiponectin Deficiency in Paneth Cells Promotes Development of Gut Organoids

**FIGURE 5:**
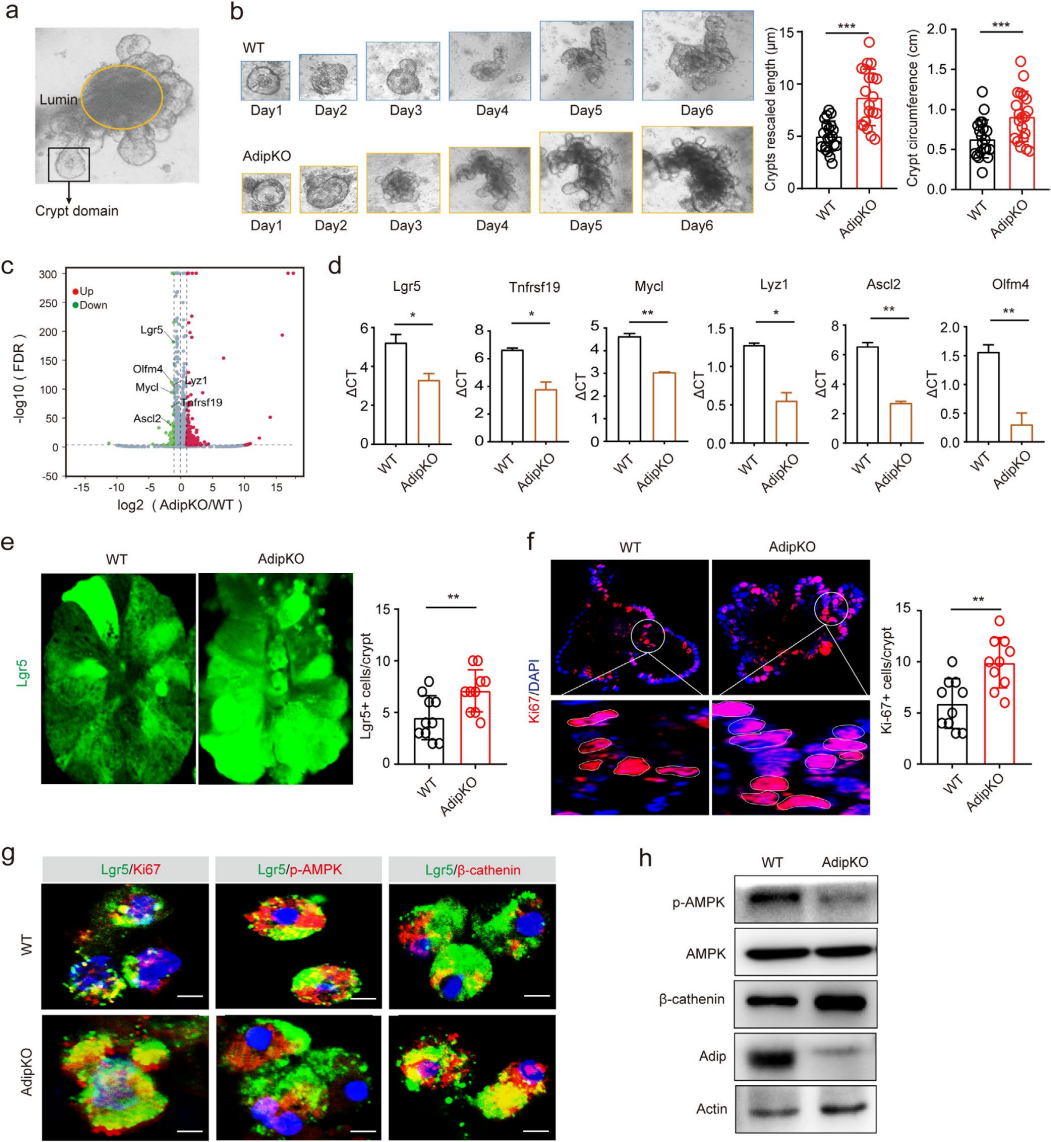
Adiponectin deficiency promotes gut organoid development. (a) A schematic illustration showing the structure of the intestinal organoids. (b) Typical bright view of in vitro cultured intestinal organoids of adip^fl/fl‐Villi‐Cre^ (AdipKO) and adip^fl/fl^ (WT) mice on day 1, day 2, day 3, day 4, day 5, and day 6. Crypt domains and circumference of per organoids were assessed. *n* = 20 organoids. (c) Volcano plot of the expression of differential genes. The data from RNA‐seq of the intestinal organoids from adip^fl/fl‐Villi‐Cre^ (AdipKO) and adip^fl/fl^ (WT) mice. (d) qRT‐PCR of lgr5, tnfrsf19, mycl, lyz1, ascl2, and olfm4 in intestinal organoids of adip^fl/fl‐Villi‐Cre^ (AdipKO) and adip^fl/fl^ (WT) mice. (e) Immunostaining of Lgr5 (green) in in vitro cultured gut organoids of adip^fl/fl‐Villi‐Cre^ (AdipKO) and adip^fl/fl^ (WT) mice at day 6. *n* = 10 crypts. (f) Immunostaining of Ki67 (red) in in vitro cultured gut organoids of adip^fl/fl‐Villi‐Cre^ (AdipKO) and adip^fl/fl^ (WT) mice at day 6, DAPI, nuclei (blue); *n* = 10 crypts; (g) Immunostaining of Lgr5/ki67, Lgr5/P‐AMPK, and Lgr5/β‐catenin in the isolated cells from in vitro cultured intestinal organoids of adip^fl/fl‐Villi‐Cre^ (AdipKO) and adip^fl/fl^ (WT) mice. (h) Immunoblotting of p‐AMPK, AMPK, β‐catenin, and adiponectin in the in vitro cultured intestinal organoids of adip^fl/fl‐Villi‐Cre^ (AdipKO) and adip^fl/fl^ (WT) mice. Student's *t*‐test, mean ± SD. *p < 0.05, **p < 0.05, ***p < 0.05.

To further elucidate the function of adiponectin derived from gut PCs, we established an intestinal organoid culture system [4]. Intriguingly, organoids generated from adip^fl/fl‐Villi‐Cre^ mice displayed accelerated growth kinetics compared to control adip^fl/fl^ organoids, with both crypt domain size and overall organoid circumference showing significant expansion (Figure 5a,b). Comprehensive transcriptomic profiling (RNA‐Seq) and subsequent qRT‐PCR validation demonstrated substantial upregulation of Lgr5^+^ stem cell markers (including lgr5, TNFrsf19, mycl, lyz1, ascl2, and olfm4 [37]) in adip^fl/fl‐Villi‐Cre^ organoids relative to controls (Figure 5c,d). Notably, expression of both adiponectin receptors (adipoR1 and adipoR2) was confirmed in these organoids (RNA‐seq data deposited in GSE269692). Consistent with the molecular signature, immunohistochemical analysis revealed a pronounced increase in both Lgr5^+^ ISCs, the fundamental units of the crypt niche responsible for self‐renewal and generation of TA progenitors [48] and proliferative cells in adip^fl/fl‐Villi‐Cre^ organoids (Figure 5e,f). These collective findings strongly suggest an inhibitory role of adiponectin in intestinal organoid development. We also observed significant downregulation of AMPK signaling coupled with elevated β‐catenin levels, a master regulator of stem cell proliferation and differentiation in adip^fl/fl‐Villi‐Cre^ organoids (Figure 5g,h), providing compelling evidence that PC‐derived adiponectin serves as a negative regulator of intestinal stem cell renewal and differentiation.

### 3.6 IAA Downregulates Adiponectin Expression

**FIGURE 6:**
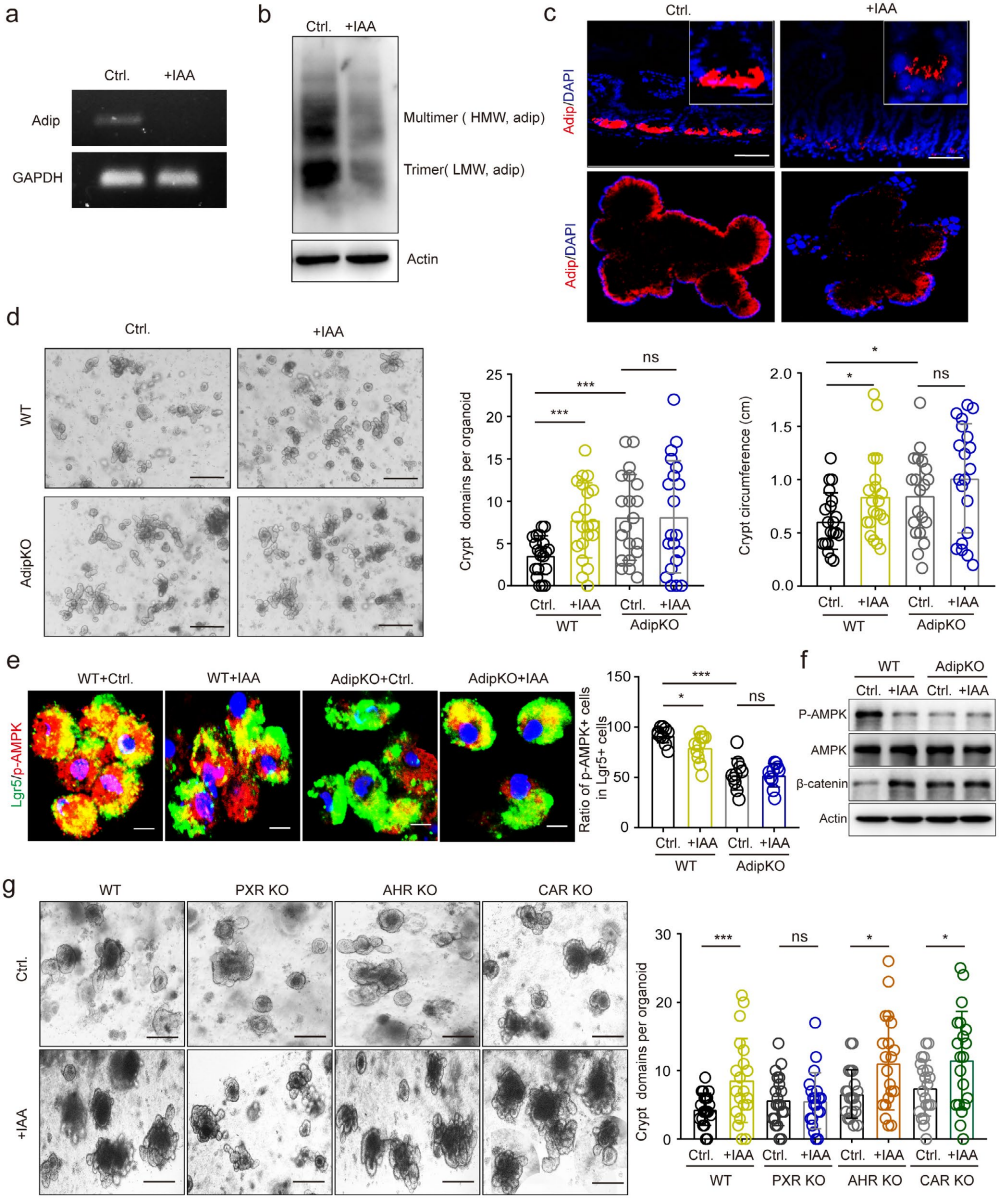
IAA downregulates the expression of adiponectin. (a) qRT‐PCR of adiponectin (Adip) in in vitro cultured gut organoids of mice with(+IAA) or without (Ctr.) IAA treatment. (b) Immunoblot of adiponectin (Adip) in in vitro cultured gut organoids of mice with(+IAA) or without (Ctr.) IAA treatment. HMW, high molecular weight; LMW, low molecular weight; Actin as the loading control. (c) Immunostaining of adiponectin (Adip) in the intestinal tissues of mice with or without IAA administration (upper) and in in vitro culture intestinal organoids (lower). Scale bar = 45 μm. (d) Bright views in in vitro cultured gut organoids from adip^fl/fl‐Villi‐Cre^ (AdipKO) and adip^fl/fl^ (WT) mice with (+IAA) or without (Ctr.) IAA. Crypt domains and circumference per organoid were assessed. *n* = 20 organoids; Scale bar = 50 μm; (e) Immunostaining of Lgr5/P‐AMPK in the isolated cells from in vitro cultured gut organoids from adip^fl/fl‐Villi‐CreT^ (AdipKO) and adip^fl/fl^ (WT) mice with (+IAA) or without (Ctr.) IAA treatment. DAPI, blue. *n* = 20 organoids; Scale bar = 10 μm; (f) Immunoblotting of p‐AMPK, AMPK, and β‐catenin in the isolated lgr5^+^cells from in vitro cultured gut organoids from adip^fl/fl‐Villi‐Cre^ (AdipKO) and dip^fl/fl^ (WT) mice with (+IAA) or without (Ctr.) IAA treatment. (g) Bright views in in vitro cultured gut organoids of WT, PXR KO (PXRKO), AHR KO (AHRKO), or CAR KO (CARKO) mice with (+IAA) or without (Ctr.) IAA treatment. Crypt domains per organoid were assessed; *n* = 20 organoids; Scale bar = 25 μm; Student's *t*‐test, mean ± SD. *p < 0.05, ***p < 0.05; Ns, no significance.

Gut microbiota regulate intestinal epithelial cell regeneration and proliferation [49]. To determine whether adiponectin‐mediated suppression of epithelial renewal and differentiation depends on the microbiota, we first examined proliferative activity and differentiation status in germ‐free (GF) mice. Notably, no differences were observed between adip^fl/fl‐Villi‐Cre^ and control adip^fl/fl^ GF mice (Figure S6), suggesting that adiponectin deficiency driven alterations in gut stem cell development require gut microbiota. Intriguingly, we discovered that IAA, a metabolite produced by Lactobacillus, suppresses adiponectin expression (Figure 6a–c). Furthermore, IAA enhanced the proliferation of gut organoids derived from adip^fl/fl^ mice but not those from adip^fl/fl‐Villi‐Cre^ mice (Figure 6d), indicating that IAA's proliferative effects are adiponectin‐dependent. Supporting this mechanism, IAA exposure significantly reduced phospho‐AMPK (p‐AMPK) and increased β‐catenin levels in adip^fl/fl^ organoids, but not in adip^fl/fl‐Villi‐Cre^ organoids (Figure 6e,f), further confirming that IAA acts through adiponectin signaling.

Previous studies have identified IAA as a potent bioactive metabolite capable of activating key nuclear receptors including pregnane X receptor (PXR), constitutive androstane receptor (CAR), and aryl hydrocarbon receptor (AHR) [50, 51]. To elucidate the potential involvement of these transcription factors in IAA‐mediated adiponectin regulation, we established intestinal organoid cultures from wild‐type (WT), AHR knockout (KO), PXR KO, and CAR KO mice. Notably, IAA treatment failed to stimulate organoid growth in PXR‐deficient cultures (Figure 6g), demonstrating that PXR activation is indispensable for IAA's proliferative effects in gut organoids. These findings collectively suggest that IAA downregulates adiponectin expression through a PXR‐dependent mechanism.

### 3.7 IAA Promotes the Renewal and Differentiation of Gut Stem Cells

**FIGURE 7:**
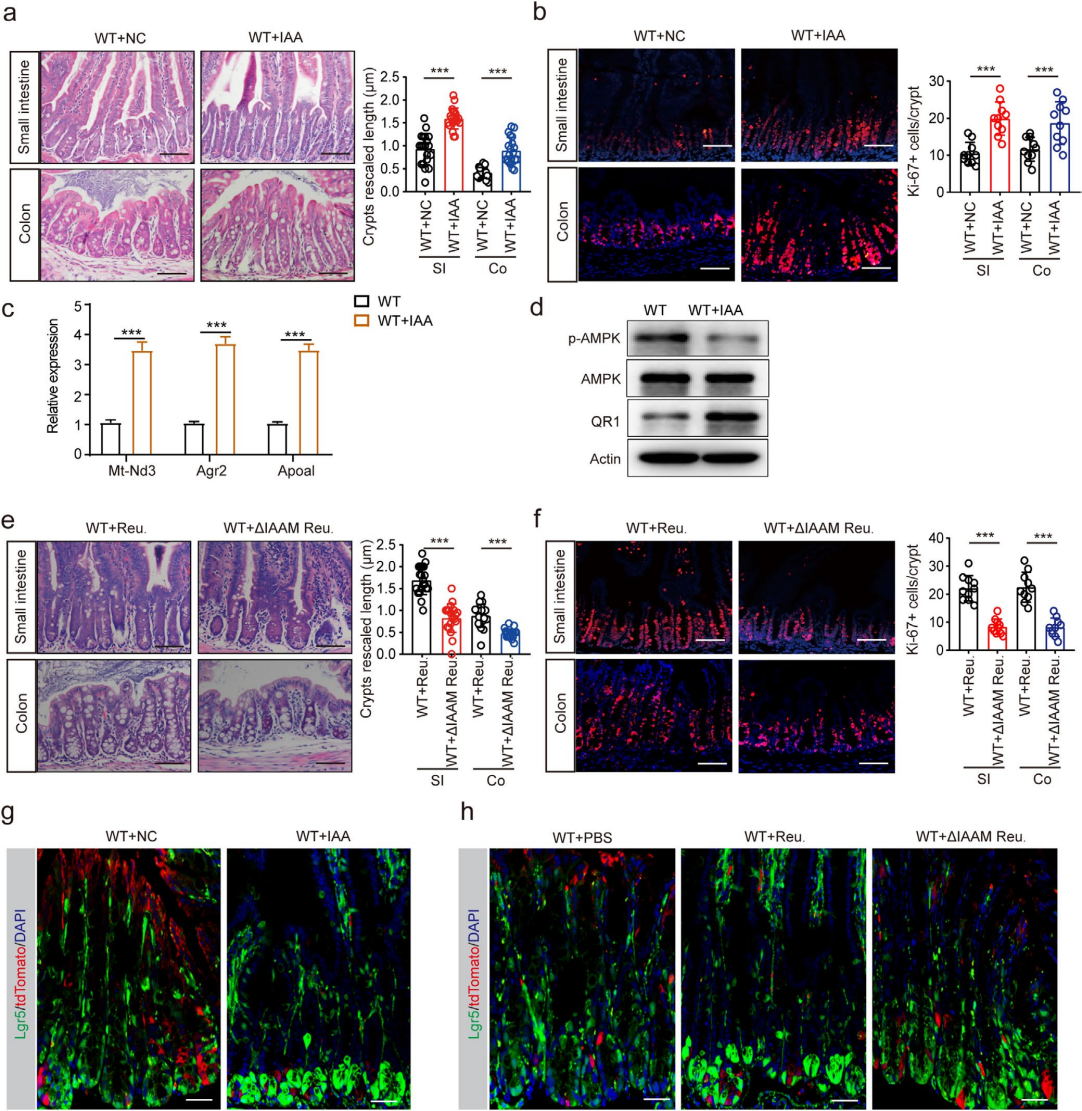
IAA promotes proliferation and differentiation of gut stem cells. (a) H&E staining of the small intestine (SI) and colon tissues (Co) of mice administered with (WT IAA) or without IAA (WT NC). *n* = 98 crypts from 5 mice per group. Scale bar = 45 μm; (b) Immunostaining of Ki67 in the small intestine (SI) and colon (Co) tissues of mice administered with (WT IAA) or without IAA (WT NC). *n* = 65 crypt‐villi units from 5 mice per group. Scale bar = 45 μm. (c) QRT‐PCR of mt‐Nd3, agr2, and apoal in the isolated ileum crypts of mice with or without IAA treatment. (d) Immunoblotting of p‐AMPK, AMPK, and β‐catenin in the isolated ileum crypts of mice with or without IAA treatment. (e) H&E staining of the small intestine (SI) and colon (Co) tissues of mice orally administered with *L. reuteri* (WT+ Reu.) or *L. reuteri* ^△iaaM^ (WT + △iaaM Reu.). *n* = 90 crypts from 5 mice per group. Scale bar = 45 μm; (f) Immunostaining of Ki67 in the small intestine (SI) and colon (Co) tissues of mice orally administered with *L. reuteri* (WT + Reu.) or *L. reuteri* ^△iaaM^ (WT + △iaaM Reu.). *n* = 70 crypt‐villi units from 5 mice per group. Scale bar = 45 μm. (g) GFP^+^ intestinal stem cells at the base of crypt in the intestine of Lgr5‐EGFP‐IRES‐CreERT2 (Lgr5‐GFP) mice administered with (+IAA) or without IAA (NC); Scale bar = 45 μm. (h) GFP^+^ intestinal stem cells at the base of crypt in the intestine of Lgr5‐EGFP‐IRES‐CreERT2 (Lgr5‐GFP) mice treated with *L. reuteri* (+Reu.) or *L. reuteri* ^△iaaM^ (+△iaaM Reu.); NC, Lgr5‐EGFP‐IRES‐CreERT2 (Lgr5‐GFP) mice administrated with PBS; DAPI for cell nuclei (blue). One typical representative of 6 mice; Scale bar = 45 μm. Student's *t*‐test in c, mean ± SD; Mann–Whitney U test in a, b, e, and f. ***p < 0.05.

To further elucidate the effects of IAA on adiponectin‐mediated inhibition of stem cell renewal and proliferation, we first examined wild‐type mice treated with or without IAA. Our observations revealed that IAA‐administered mice exhibited significantly smaller intestinal crypts compared to control mice (Figure 7a,b). Molecular analysis demonstrated upregulated expression of mt‐Nd3, agr2, and apoal in the ileum crypts of IAA‐treated mice (Figure 7c). Furthermore, the ileum crypts from IAA‐treated WT mice showed markedly reduced p‐AMPK levels and increased β‐catenin expression compared to untreated controls (Figure 7d). To specifically assess IAA's role, we used a mutant strain of *Lactobacillus reuteri* ( *L. reuteri* ^δiaam^) that lacks IAA production capability [28]. Intriguingly, mice colonized with *L. reuteri* ^δiaam^ developed smaller crypts than those receiving wild‐type *L. reuteri* (Figure 7e). Notably, wild‐type *L. reuteri* administration resulted in crypt enlargement compared to untreated mice (Figure S7a). Immunohistochemical analysis revealed significantly more Ki67^+^ proliferative cells in the gut epithelium of wild‐type *L. reuteri* –treated mice, but not in those receiving the mutant strain (Figure 7f). To investigate the potential involvement of the PXR pathway, we examined PXR knockout mice. Interestingly, *L. reuteri* administration failed to induce significant crypt changes in PXR KO mice (Figure S7b), suggesting PXR's essential role in this process. Using Lgr5EGFP‐IRES‐creERT2 reporter mice [52, 53] to track ISCs, we observed substantial increases in Lgr5^+^ stem cell populations following IAA or wild‐type *L. reuteri* treatment, but not with *L. reuteri* ^δiaam^ (Figure 7g,h). These findings collectively demonstrate that gut microbiota‐derived IAA promotes crypt cell enrichment through an adiponectin‐dependent mechanism.

## 4 Discussion

Here, we reveal a critical role of PC‐derived adiponectin in regulating intestinal stem cell renewal and differentiation to maintain gut epithelial homeostasis. Importantly, we identify IAA, a gut microbiota‐derived metabolite, as a key modulator of adiponectin expression in PCs. Together, our findings delineate a previously unrecognized gut microbiota‐Paneth cell‐intestinal stem cell axis that is fundamental for proper epithelial renewal and homeostasis in the intestinal tract.

We demonstrate that adiponectin, secreted by PCs residing at the base of intestinal crypts, serves as a key regulator that suppresses the proliferation and differentiation of ISCs, thereby maintaining epithelial homeostasis in the gut. In both the small intestine and colon, PCs form the essential niche microenvironment for Lgr5^+^ stem cells within the crypts [5]. While these specialized cells are well known for promoting ISC renewal and differentiation through niche factors like Wnt3 [5], our findings reveal their equally crucial role in constraining excessive stem cell proliferation. These regulatory functions position PCs as central players in orchestrating the delicate balance between stem cell maintenance and differentiation that underpins gut epithelial homeostasis.

PC‐derived adiponectin suppresses ISC regeneration, proliferation, and differentiation via the AMPK pathway, thereby inhibiting key signaling systems that regulate ISC self‐renewal and multipotency [12, 54]. In line with this suppression, adip^fl/fl‐Villi‐Cre^ mice exhibit compensatory upregulation of multiple signaling pathways in stem and TA cells. Notably, the PI3K/Akt/mTOR pathway, a central regulator of ISC stemness maintenance, proliferation, differentiation, epithelial‐to‐mesenchymal transition (EMT), migration, and autophagy [55] is enhanced. This pathway plays a critical role in ISC‐mediated epithelial regeneration and repair [56] and interacts with several other cascades, including androgen receptor (AR), MAPK, and Wnt signaling [57]. Wnt/β‐catenin signaling, the primary activator of ISCs [58], is also elevated in the stem and TA cells of these mice. This pathway drives cyclin D1 expression and cell cycle activation, and its stimulation can restore epithelial regeneration [59]. Furthermore, these cells display upregulated metabolic pathways, consistent with AMPK's role as a master regulator of cellular energy homeostasis [60]. Intriguingly, ISC maintenance can persist even in the absence of PCs [61, 62], suggesting alternative mechanisms for stem cell regulation.

Our findings further demonstrate that gut microbiota‐derived IAA, produced by *L. reuteri* , enhances ISC enrichment and proliferation by suppressing adiponectin expression in PCs. The gut microbiota plays a pivotal role in maintaining intestinal epithelial homeostasis, with accumulating evidence highlighting the interplay between microbial metabolites and IEC development. Key metabolites, including bile acids, short‐chain fatty acids (SCFAs), tryptophan derivatives, and lactate, have been shown to promote ISC‐driven epithelial regeneration [63, 64, 65]. Indole and its microbial metabolites also exhibit protective effects against gut injury [66]. The exogenous IAA administration has been reported to alleviate intestinal ischemia/reperfusion damage [67].

Since gut microbiota‐derived indole‐3‐acetic acid (IAA) can downregulate adiponectin expression in Paneth cells, thereby promoting intestinal stem cell (ISC) renewal and proliferation, enhancing intestinal epithelial repair in patients with conditions such as inflammatory bowel disease (IBD) may be achieved through two potential therapeutic strategies: modulating the gut microbiota or directly targeting adiponectin in Paneth cells.

## Author Contributions

R.Y. designed the research and wrote the paper; H.L. conducted in vivo experiments; X.S. conducted in vitro culture organoid; J.W. analyzed data and conducted some in vivo and in vitro experiments; M.J. conducted some in vitro experiments; Y.Z. offered assistance for the animal experiments. All authors read and approved the final manuscript.

## Ethics Statement

The animal study protocol was approved by the Institutional Animal Care and Use Committee of Nankai University (protocol code NK2020121 and date of approval December 1, 2020).

## Conflicts of Interest

The authors declare no conflicts of interest.

## Supporting information


**Figure S1:** Expression of adiponectin in the bottom of crypts. Immunostaining of adiponectin (Adip)/lysozyme, and adiponectin (Adip)/Lgr5 in the small intestinal (SI) (a) and colonic (Co) (b) tissues of adip^fl/fl‐Villi‐Cre^ (Ad KO) and adip^fl/fl^ (WT) mice. Scale bar = 45 μm.
**Figure S2:** Identification of different ileum cell clusters in adip^fl/fl‐Villi‐Cre^ (QKO) and adip^fl/fl^ (QNC) mice. (a) UMAP of ileum cell clusters in adip^fl/fl‐Villi‐Cre^ (QKO) and adip^fl/fl^ (QNC) mice; (b) Gene expression in different ileum cell clusters of adip^fl/fl‐Villi‐Cre^ (QKO) and adip^fl/fl^ (QNC) mice.
**Figure S3:** Expression of adipR1 and adipR2 in the intestinal stem cells. Slides of intestine and colon tissues were stained using anti‐adipR1 (Red)/LGR5 (Green) or anti‐adipR2 (Red)/LGR5 (Green) antibody. Scale bar = 45 μm.
**Figure S4:** Proliferation of gut stem and TA cells in AdipR1 KO and WT mice. H/E staining in duodenum (Duo), jejunum (Jej), and ileum (Ileum) (a) and in proximal colon (PC), middle colon (MC), and distal colon (DC) (b) of AdipR1 KO and WT mice; Crypt rescaled length was analyzed; *n* = 105 crypts in 5 mice. Ki67 staining in duodenum (Duo), jejunum (Jej), and ileum (ileu) (a) and in proximal colon (PC), middle colon (MC), and distal colon (DC) (b) of AdipR1 KO and WT mice; Ki67+ cells were analyzed; *n* = 66 crypt‐villus units in 5 mice. Mann–Whitney U test; *p < 0.05, **p < 0.05, ***p < 0.05; Scale bar = 45 μm.
**Figure S5:** Single‐cell transcription analyses of TA cluster. (a) Gene expression of ileum TA cluster in adip^fl/fl‐Villi‐Cre^ (QKO) and adip^fl/fl^ (QNC) mice; (b) KEGG analyses of intestinal TA cluster in adip^fl/fl‐Villi‐Cre^ (QKO) and adip^fl/fl^ (QNC) mice.
**Figure S6:** Proliferation of gut stem and TA cells in newborn germ‐free mice. (a) H/E (upper) and Ki67 (lower) staining in duodenum (Duo), jejunum (jej), and ileum (ileum) of newborn adip^fl/fl‐Villi‐Cre^ (AdipKO) and adip^fl/fl^ (WT) germ‐free mice. Crypt rescaled high and Ki67^+^ cells were analyzed; *n* = 88 crypts for crypt rescaled length in 5 mice; *n* = 60 crypt‐villus units for Ki67 cells in 5 mice. (b) H/E (upper) and Ki67 (lower) staining in proximal colon (PC), middle colon (MC), and distal colon (DC) of newborn adip^fl/fl‐Villi‐Cre^ (AdipKO) and adip^fl/fl^ (WT) germ‐free mice. Crypt rescaled length and Ki67^+^ cells were analyzed. *n* = 108 crypts for crypt rescaled length in 5 mice; *n* = 65 crypt‐villus units for Ki67 cells in 5 mice. Scale bar = 45 μm；
Mann–Whitney U test; Ns, no significance.
**Figure S7:** Proliferation of gut stem and TA cells in mice orally administered with or without *L. reuteri* . (a) H/E staining in duodenum (Duo), jejunum (Jej), and ileum (Ileu, upper) and in proximal colon (PC), middle colon (MC), and distal colon (DC) of mice with (+LAC) or without *L. reuteri* . Crypt rescaled high were analyzed. *n* = 102 crypts for crypt rescaled length in 5 mice. (b) H/E staining in duodenum (Duo), jejunum (Jej) and ileum (Ileu, upper) and in proximal colon (PC), middle colon (MC), and distal colon (DC) of PXR KO (PXRKO) mice with (PXRKO+LAC) or without *L. reuteri* . Crypt rescaled length were analyzed. *n* = 95 crypts for crypt rescaled length in 5 mice. Scale bar = 45 μm;
Mann–Whitney U test; *p < 0.05, **p < 0.05, ***p < 0.05; Ns, no significance.
**Table S1:** Reagents and oligos used in this study.

## Data Availability

The data that support the findings of this study are available from the corresponding author upon reasonable request. GEO accession number: RNA‐seq: GSE269692; sc‐RNA‐seq: GSE270099. All data generated or analyzed during this study are available from the corresponding author on reasonable request.
