# Supplementary material for: Adiponectin Regulated by Indole‐3‐Acetic Acid in Paneth Cells Controls Renewal and Differentiation of Gut Stem Cells
Source: FASEB J. 2025 Oct 6;39(19):e71101. doi: 10.1096/fj.202501229RR (PMC12499647; doi:10.1096/fj.202501229RR)
Supplement: Supplementary file 1 — Figure S1: Expression of adiponectin in the bottom of crypts. Immunostaining of adiponectin (Adip)/lysozyme, and adiponectin (Adip)/Lgr5 in the small intestinal (SI) (a) and colonic (Co) (b) tissues of adipfl/fl‐Villi‐Cre (Ad KO) and adipfl/fl (WT) mice. Scale bar = 45 μm. Figure S2: Identification of different ileum cell clusters in adipfl/fl‐Villi‐Cre (QKO) and adipfl/fl (QNC) mice. (a) UMAP of ileum cell clusters in adipfl/fl‐Villi‐Cre (QKO) and adipfl/fl (QNC) mice; (b) Gene expression in different ileum cell clusters of adipfl/fl‐Villi‐Cre (QKO) and adipfl/fl (QNC) mice. Figure S3: Expression of adipR1 and adipR2 in the intestinal stem cells. Slides of intestine and colon tissues were stained using anti‐adipR1 (Red)/LGR5 (Green) or anti‐adipR2 (Red)/LGR5 (Green) antibody. Scale bar = 45 μm. Figure S4: Proliferation of gut stem and TA cells in AdipR1 KO and WT mice. H/E staining in duodenum (Duo), jejunum (Jej), and ileum (Ileum) (a) and in proximal colon (PC), middle colon (MC), and distal colon (DC) (b) of AdipR1 KO and WT mice; Crypt rescaled length was analyzed; n = 105 crypts in 5 mice. Ki67 staining in duodenum (Duo), jejunum (Jej), and ileum (ileu) (a) and in proximal colon (PC), middle colon (MC), and distal colon (DC) (b) of AdipR1 KO and WT mice; Ki67+ cells were analyzed; n = 66 crypt‐villus units in 5 mice. Mann–Whitney U test; *p < 0.05, **p < 0.05, ***p < 0.05; Scale bar = 45 μm. Figure S5: Single‐cell transcription analyses of TA cluster. (a) Gene expression of ileum TA cluster in adipfl/fl‐Villi‐Cre (QKO) and adipfl/fl (QNC) mice; (b) KEGG analyses of intestinal TA cluster in adipfl/fl‐Villi‐Cre (QKO) and adipfl/fl (QNC) mice. Figure S6: Proliferation of gut stem and TA cells in newborn germ‐free mice. (a) H/E (upper) and Ki67 (lower) staining in duodenum (Duo), jejunum (jej), and ileum (ileum) of newborn adipfl/fl‐Villi‐Cre (AdipKO) and adipfl/fl (WT) germ‐free mice. Crypt rescaled high and Ki67+ cells were analyzed; n = 88 crypts for crypt resc [file FSB2-39-e71101-s001.docx]

**Adiponectin regulated by indole-3-acetic acid in Paneth cells controls renewal and differentiation of gut stem cells**

Hang Liu^1^, Xiaomin Su^1^, Juanjuan Wang^1^, Mengli Jin^1^, Yuan Zhang^1^ & Rongcun Yang^1,2,3,4^

**
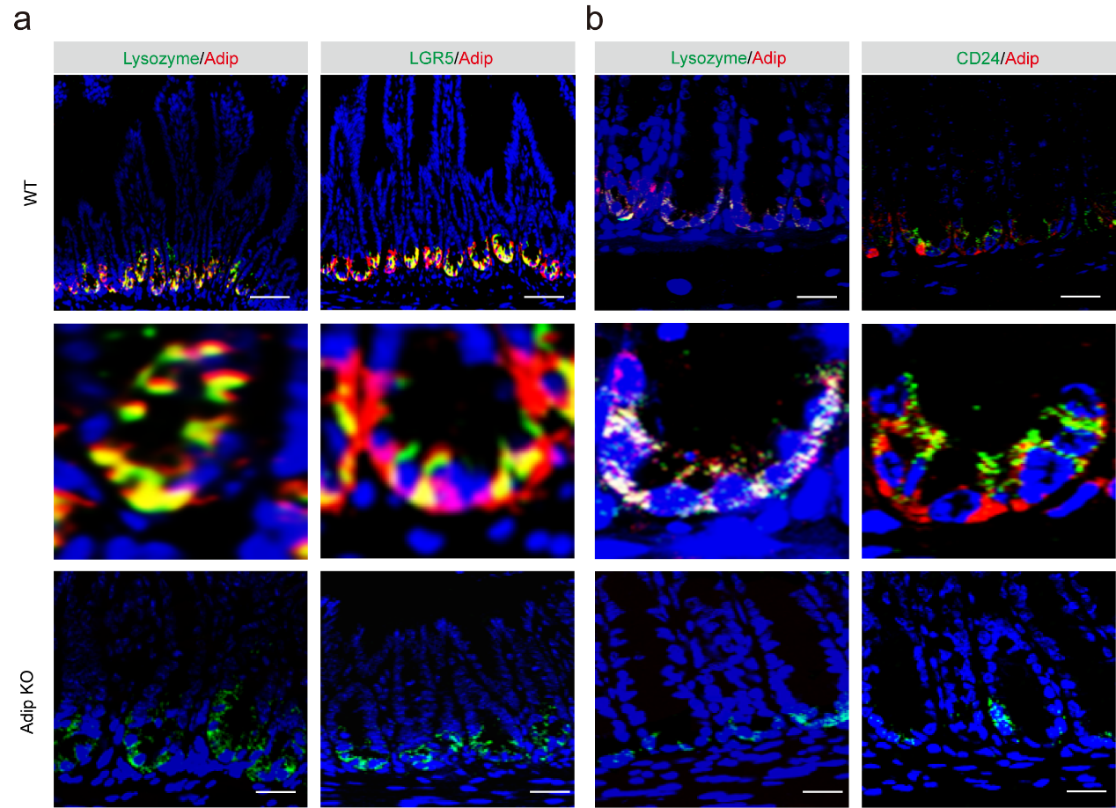
**

FIGURE S1. Expression of adiponectin in the bottom of crypts.

Immunostaining of adiponectin (Adip)/lysozyme, and adiponectin (Adip)/Lgr5 in the small intestinal (SI) (a) and colonic (Co) (b) tissues of **adip^fl/fl-Villi-Cre^** (Ad KO) and adip^fl/fl^ (WT) mice. Scale bar=45 μm.

**
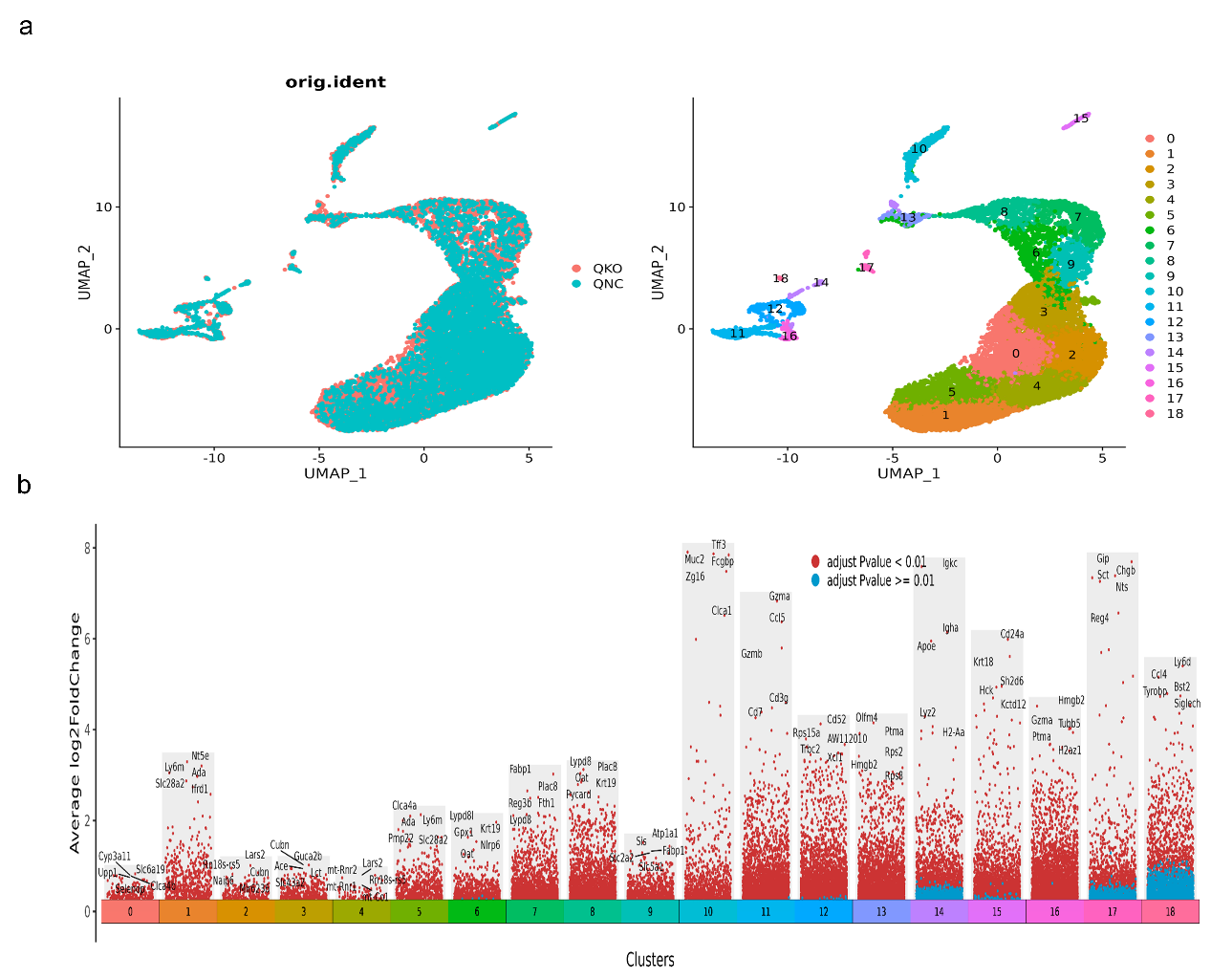
**

FIGURE S2. Identification of different ileum cell clusters in **adip^fl/fl-Villi-Cre^** (QKO) and adip^fl/fl^ (QNC) mice. (a) UMAP of ileum cell clusters in **adip^fl/fl-Villi-Cre^** (QKO) and adip^fl/fl^ (QNC) mice; (b) Gene expression in different ileum cell clusters of **adip^fl/fl-Villi-Cre^** (QKO) and adip^fl/fl^ (QNC) mice.


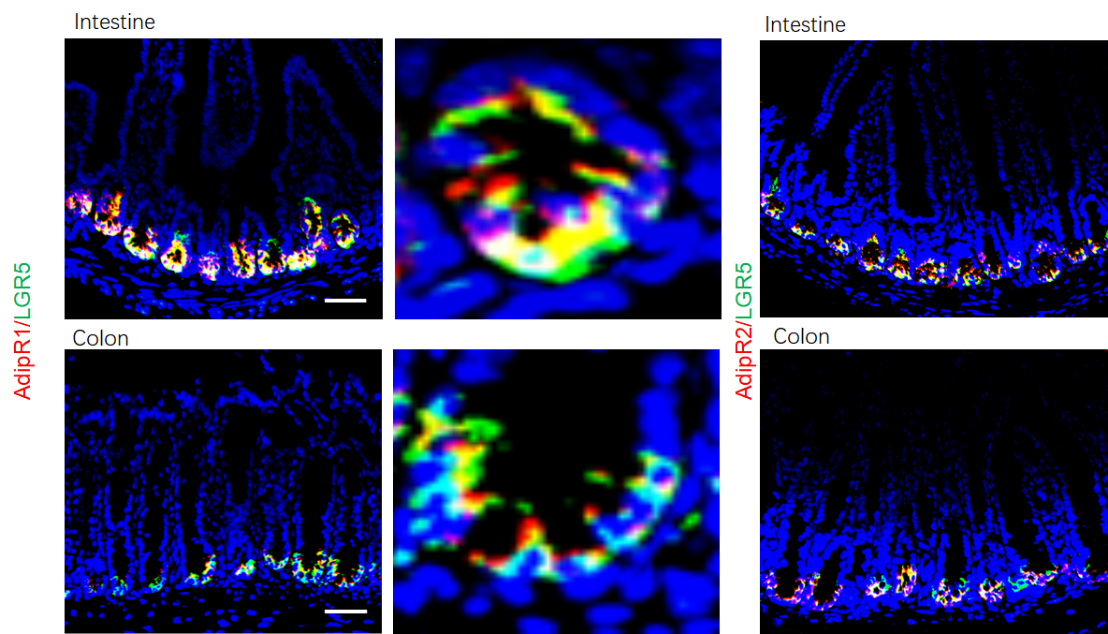


FIGURE S3. Expression of adipR1 and adipR2 in the intestinal stem cells. Slides of intestine and colon tissues were stained using anti-adipR1 (Red)/LGR5 (Green) or anti-adipR2 (Red)/LGR5 (Green) antibody. Scale bar=45 μm.

**
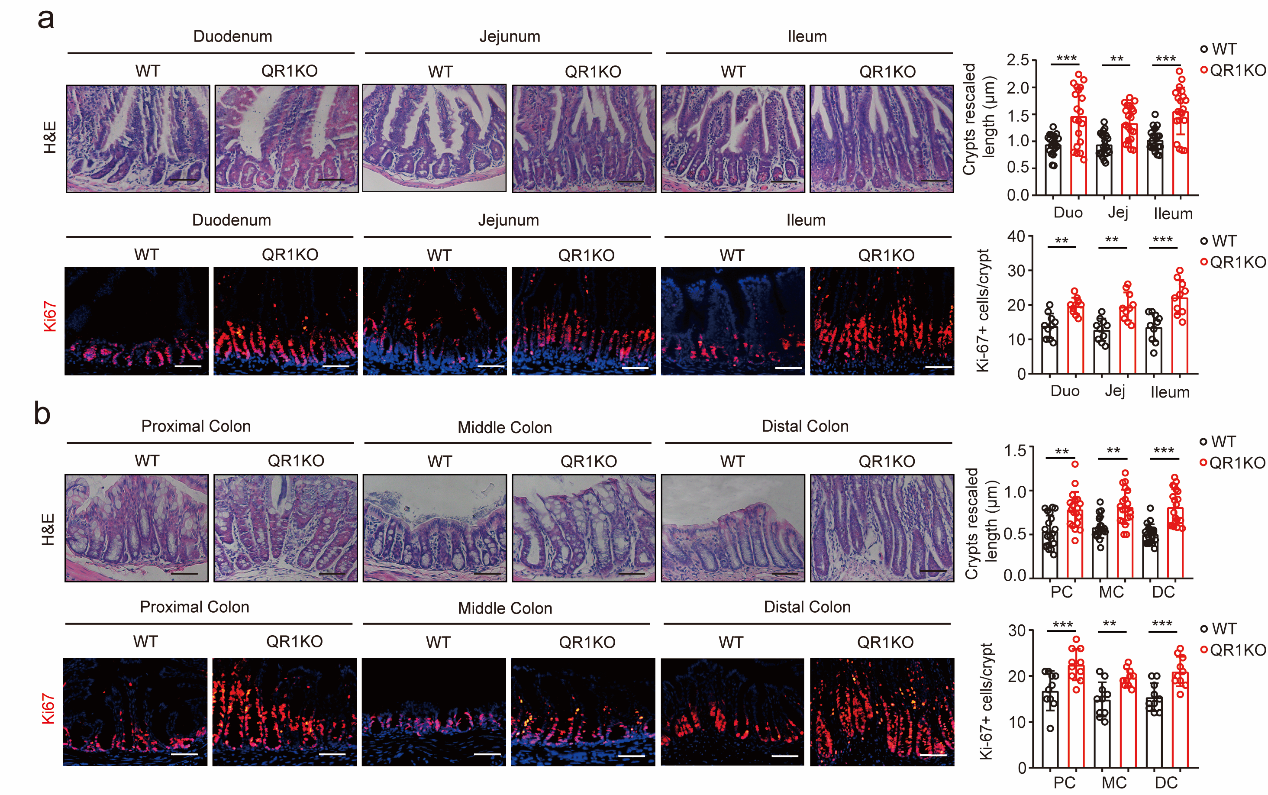
**

FIGURE S4. Proliferation of gut stem and TA cells in AdipR1 KO and WT mice.

H/E staining in duodenum (Duo), jejunum (Jej) and ileum (Ileum) (a) and in proximal colon (PC), middle colon (MC) and distal colon (DC) (b) of AdipR1 KO and WT mice; Crypt rescaled length was analyzed; n=105 crypts in 5 mice. Ki67 staining in duodenum (Duo), jejunum (Jej) and ileum (ileu) (a) and in proximal colon (PC), middle colon (MC) and distal colon (DC) (b) of AdipR1 KO and WT mice; Ki67+ cells were analyzed; n=66 crypt-villus units in 5 mice. Mann–Whitney U test; *p<0.05, **p<0.05, ***p<0.05; Scale bar=45 μm.

**
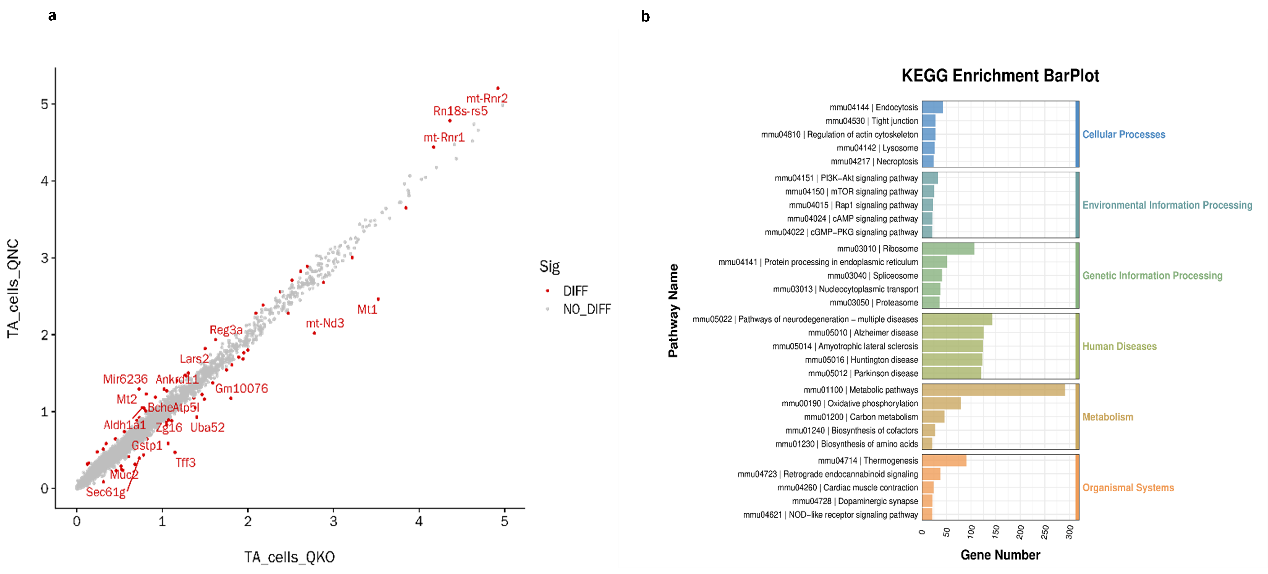
**

FIGURE S5. Single cell transcription analyses of TA cluster.

1. Gene expression of ileum TA cluster in **adip^fl/fl-Villi-Cre^** (QKO) and adip^fl/fl^ (QNC) mice; (b) KEGG analyses of intestinal TA cluster in **adip^fl/fl-Villi-Cre^** (QKO) and adip^fl/fl^ (QNC) mice.

**
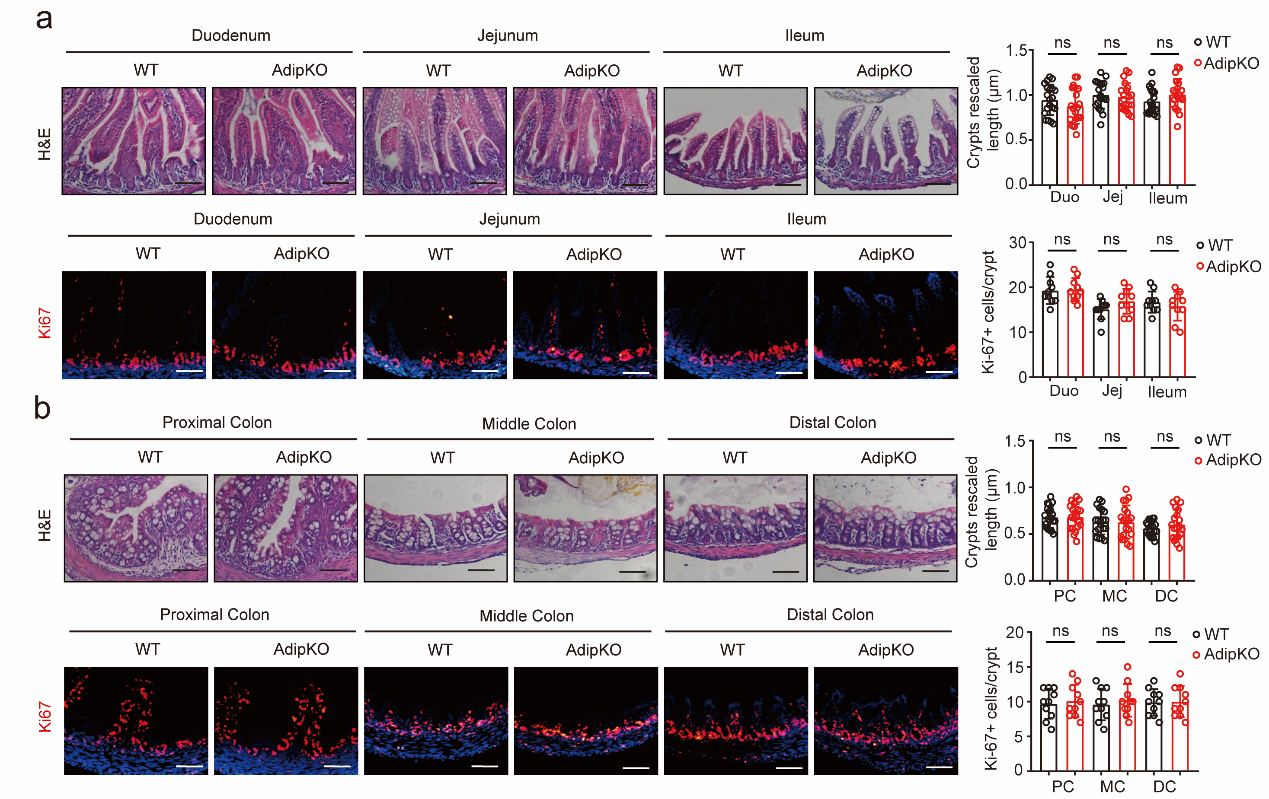
**

FIGURE S6. Proliferation of gut stem and TA cells in new-born germ-free mice.

(a) H/E (upper) and Ki67 (lower) staining in duodenum (Duo), jejunum (jej) and ileum (ileum) of new-born **adip^fl/fl-Villi-Cre^** (AdipKO) and adip^fl/fl^ (WT) germ-free mice. Crypt rescaled high and Ki67^+^ cells were analyzed; n=88 crypts for crypt rescaled length in 5 mice; n=60 crypt-villus units for Ki67 cells in 5 mice. (b) H/E (upper) and Ki67 (lower) staining in proximal colon (PC), middle colon (MC) and distal colon (DC) of new-born **adip^fl/fl-Villi-Cre^** (AdipKO) and adip^fl/fl^ (WT) germ-free mice. Crypt rescaled length and Ki67^+^ cells were analysed. n=108 crypts for crypt rescaled length in 5 mice; n= 65 crypt-villus units for Ki67 cells in 5 mice. Scale bar=45 μm；

Mann–Whitney U test; Ns, no significance.


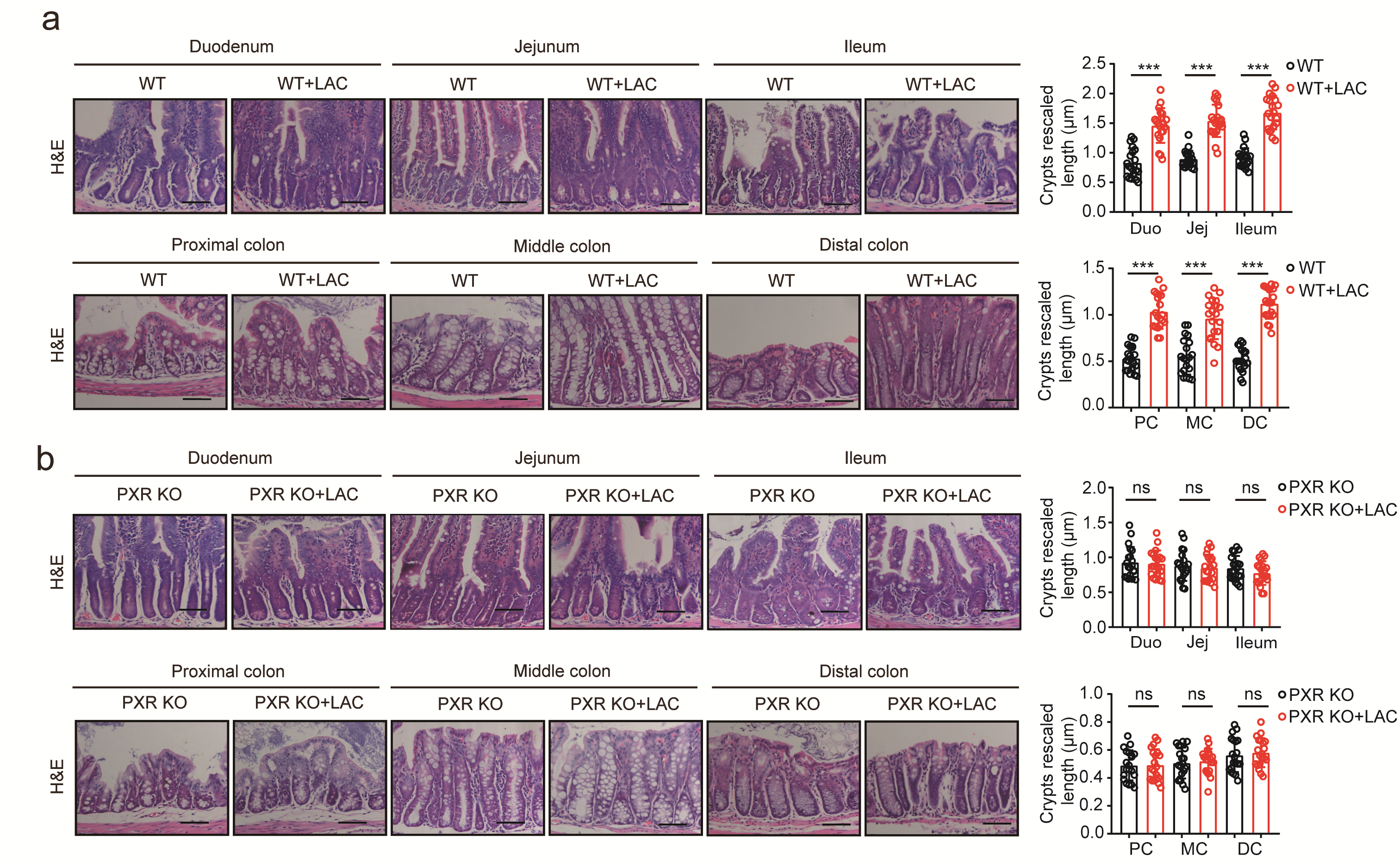


FIGURE S7. Proliferation of gut stem and TA cells in mice orally administered with or without L. reuteri. (a) H/E staining in duodenum (Duo), jejunum (Jej) and ileum (Ileu, upper) and in proximal colon (PC), meddle colon (MC) and distal colon (DC) of mice with (+LAC) or without L. reuteri. Crypt rescaled high were analysed. n=102 crypts for crypt rescaled length in 5 mice. (b) H/E staining in duodenum (Duo), jejunum (Jej) and ileum (Ileu, upper) and in proximal colon (PC), meddle colon (MC) and distal colon (DC) of PXR KO (PXRKO) mice with (PXRKO+LAC) or without L. reuteri. Crypt rescaled length were analyzed. n=95 crypts for crypt rescaled length in 5 mice. Scale bar=45 μm;

Mann–Whitney U test; *p<0.05, **p<0.05, ***p<0.05; Ns, no significance.

**Table S1.** **Reagents and oligoes used in this study.**

| **REAGENT or RESOURCE** | **SOURCE** | **IDENTIFIER** |  |
| --- | --- | --- | --- |
| Antibodies for western blot and immunostaining | | |  |
| β-Actin Antibody | Santa Cruz | Cat:sc-47778 |  |
| Anti- Adiponectin Antibody | Abcam | Cat: ab181281 |  |
| Anti-AMPK Antibody | Abcam | Cat: ab131512 |  |
| Anti- p-AMPK Antibody | Abcam | Cat: ab23875 |  |
| Anti-β-catenin Antibody | Cell Signaling Technology | Cat: 8480S |  |
| mTOR Monoclonal antibody | Proteintech | Cat: 66888-1-Ig RRID: AB_2882219 |  |
| Phospho-mTOR Monoclonal antibody | Proteintech | Cat: 10176-2-AP RRID: AB_2224574 |  |
| AKT Polyclonal antibody | Proteintech | Cat: 10176-2-AP RRID: AB_2224574 |  |
| Phospho-AKT (Ser473) Monoclonal antibody | Proteintech | Cat: 66444-1-Ig RRID: AB_2782958 |  |
| PIK3CA Recombinant antibody | Proteintech | Cat: 82796-4-RR RRID: AB_3086541 |  |
| PI3 Kinase p85 Antibody | Cell Signaling Technology | Cat: 4292S RRID: AB_329869 |  |
| Phospho-PI3K p85/p55 (Tyr458, Tyr199) Polyclonal Antibody | Invitrogen | Cat: PA5-17387 RRID: AB_10985894 |  |
| AMPK alpha Polyclonal Antibody | Invitrogen | Cat: PA5-105297 RRID: AB_2853890 |  |
| Phospho-AMPKα Rabbit mAb | Invitrogen | Cat: PA5-37821 RRID: AB_2554429 |  |
| Anti- Adiponectin Receptor1 Antibody | Abcam | Cat: ab126611 |  |
| Anti- Adiponectin Receptor2 Antibody | Abcam | Cat: ab231051 |  |
| Anti-GAPDH Antibody | Abclonal | Cat: A19056 |  |
| Anti-Lysozyme Antibody | Abcam | Cat: ab108508 |  |
| Anti-LGR5 Antibody | Thermo Fisher Scientific | Cat:MA5-25644 |  |
| Anti-Ki67 Antibody | Abcam | Cat: ab15580 |  |
| Anti-CD24 Antibody | Thermo Fisher Scientific | Cat:MA5-11828 |  |
| FITC-Goat Anti-Rat IgG（H+L） | Proteintech | Cat: SA00003-11 |  |
| TRITC-Goat Anti-Rat IgG（H+L） | Proteintech | Cat: SA00007-7 |  |
| Goat Anti-Mouse IgG H&L (Alexa Fluor® 488) | Abcam | Cat: ab150113 |  |
| Goat Anti-Mouse IgG H&L (Alexa Fluor® 594) | Abcam | Cat: ab150116 |  |
| Goat Anti-Rabbit IgG H&L (Alexa Fluor® 488) | Abcam | Cat: ab150077 |  |
| Goat Anti-Rabbit IgG H&L (Alexa Fluor® 594) | Abcam | Cat: ab150080 |  |
| （TRITC）-conjugated Rabbit Anti-Goat IgG(H+L) | Proteintech | Cat: SA00007-4 |  |
| Rabbit Anti-Goat IgG H&L / HRP antibody | Bioss | Cat: bs-0294R |  |
| Bacteria strain |  |  |  |
| *Lactobacillus reuteri* | ATCC23272 | |  |
| Chemicals | | |  |
| 3-Indoleacetic acid | MCE | Cat: HY-18569 |  |
| Recombinant mouse adiponectin | R＆D systems | Cat: 5095-AC-050 |  |
| Y-27632 | MCE | Cat: HY-10071 |  |
| Dextran sodium sulfate | MP Biomedicals | Cat: 160110 |  |
| IntestiCult™ OGM Human Basel Medium | Stemcell technologies | Cat: 100-0190 |  |
| Organoid Supplement | Stemcell technologies | Cat: 100-0191 |  |
| EGF | Gibco | Cat: PHG0313 |  |
| Noggin | Gibco | Cat: 120-10C |  |
| R-spondin | Gibco | Cat: 120-38 |  |
| HEPES | Gibco | Cat: 15630080 |  |
| Trizol | Life Technologies | Cat: 15596026 |  |
| β-mercaptoethanol | Gibco | Cat: 21985023 |  |
| FBS | Gibco | Cat:10099141 |  |
| DAPI | S ThemBiotech | Cat:0100-20 |  |
| Collagenase IV | Sigma-Aldrich | Cat: C5138 |  |
| Dnase I | Solarbio | Cat: D8071 |  |
| TrypLE^TM^ Express | Gibco | Cat: 12604013 |  |
| Dispase | Corning | Cat: 354235 |  |
| DMEM | Gibco | Cat:11965118 |  |
| HBSS | Gibco | Cat:14170161 |  |
| Pecoll | Solarbio | Cat: P8370 |  |
| PMA | Sigma-Aldrich | Cat: 79346 |  |
| Ionomycin | Sigma-Aldrich | Cat: 19657 |  |
| GolgiStop | BD Biosciences | Cat: 554724 |  |
| EDTA | Sigma-Aldrich | Cat: 798681 |  |
| Gentle Cell Dissociation Reagent | STEMCELL | Cat: 07174 |  |
| Matrigel | Corning | Cat:356231 |  |
| PMSF | Solarbio | Cat: P0100 |  |
| DMEM/F12 | STEMCELL | Cat:36254 |  |
| QIAquick PCR Purification Kit | Qiagen | Cat:28104 |  |
| QuantiTect SYBR Green PCR Master Mix | Qiagen | Cat:208052 |  |
| ECL chemiluminescence | Absin | Cat: abs920 |  |
| Protease Inhibitor Cocktail | Sigma | Cat: P8340 |  |
| Maconkey | Solarbio | Cat: LA8200 |  |
| Maconkey Agar | Solarbio | Cat: M8560 |  |
| Native lysis Buffer | Solarbio | Cat: R0030 |  |
| Streptomycin Sulfate | Solarbio | Cat: S8290 |  |
| Ampicillin | Sigma | Cat: 69-52-3 |  |
| Vancomycin | Sigma | Cat: 1404-93-9 |  |
| Neomycin sulfate | Sigma | Cat:1405-10-3 |  |
| Metronidazole | Sigma | Cat: 443-48-1 |  |
| Erythrocyte lysis solution | MACS | Cat: 130-094-183 |  |
| Dead Cell Removal MicroBeads | MACS | Cat: 130-090-101 |  |
| MRS broth | Basebio | Cat: BS1138 |  |
| Oligonucleotides for qRT-PCR | | | |
| Murine Adiponectin FW | BGI | 5’- CTTGGTCCTAAGGGTGAGACA -3’ | |
| Murine Adiponectin REV | BGI | 5’- GAGCGATACACATAAGCGGCT -3’ | |
| Murine ki67 FW | BGI | 5’- CTGATGTTGTAAAACTTGGCGT -3’ | |
| Murine ki67 Rev | BGI | 5’- ATTGTGAAGATTGCTTGTGGGT -3’ | |
| Murine Lgr5 FW | BGI | 5’- AAGTACAATGCCTCTCCCCTC -3’ | |
| Murine Lgr5 REV | BGI | 5’- CCAGCTCTCCTTTCTCCAAAC -3’ | |
| Murine Lysozyme FW | BGI | 5’- TCCTGACTCTGGGACTCCTCC -3’ | |
| Murine Lysozyme REV | BGI | 5’- GTGCTTTGGTCTCCACGGTTG -3’ | |
| Murine Mt-Nd3 FW | BGI | 5’- CTGTACTCAGAAAAAGCAAA -3’ | |
| Murine Mt-Nd3 REV | BGI | 5’- AGGTCAAATAATAGAAATGT -3’ | |
| Murine Agr2 FW | BGI | 5’- AAATTGGCAGAGCAGTTTGTT -3’ | |
| Murine Agr2 REV | BGI | 5’- TAGAGCCGGTTTGAGTATCGT -3’ | |
| Murine Apoa1 FW | BGI | 5’- GCCCATTGACTCGGGACTTCT -3’ | |
| Murine Apoa1 REV | BGI | 5’- CATCCTCTTTCCATTTCTTCT -3’ | |
| Murine GAPDH FW | BGI | TCAACGGCACAGTCAAGG |  |
| Murine GAPDH Rev | BGI | TACTCAGCACCGGCCTCA |  |
| Murine Tnfrsf19 FW | BGI | TTCTGTGGGGGACACGATG |  |
| Murine Tnfrsf19 Rev | BGI | AGAAAATTCAGCGCAGATGGAA |  |
| Murine Mycl FW | BGI | GCACTTCCATATCTCTATCCAC |  |
| Murine Mycl Rev | BGI | ACAATCTCTTCTTCCTCCTCTT |  |
| Murine Ascl2 FW | BGI | AAGCACACCTTGACTGGTACG |  |
| Murine Ascl2 Rev | BGI | AAGTGGACGTTTGCACCTTCA |  |
| Murine OLFM4 FW | BGI | CAGCCACTTTCCAATTTCACTG |  |
| Murine OLFM4 Rev | BGI | GCTGGACATACTCCTTCACCTTA |  |
